# An overview of *Agaricus* section *Hondenses* and *Agaricus* section *Xanthodermatei* with description of eight new species from Pakistan

**DOI:** 10.1038/s41598-021-92261-5

**Published:** 2021-06-18

**Authors:** Hira Bashir, Jie Chen, Sana Jabeen, Sadiq Ullah, Junaid Khan, Abdul Rehman Niazi, Mingzhe Zhang, Abdul Nasir Khalid, Luis Alberto Parra, Philippe Callac

**Affiliations:** 1 Fungal Biology and Systematics Lab, Department of Botany, University of the Punjab, Quaid-e-Azam Campus, Lahore, 54590 Pakistan; 2 INRAE, MycSA, CS 20032, 33882 Villenave d’Ornon, France; 3 Facultad de Ciencias Biológicas y Agropecuarias, Peñuela, Universidad Veracruzana, Amatlán de Los Reyes, 94945 Veracruz, Mexico; 4 Division of Science and Technology, Department of Botany, University of Education, Township, Lahore, Pakistan; 5 Department of Botany, Hazara University Mansehra, Khyber Pakhtunkhwa, 21300 Pakistan; 6 Center for Plant Sciences and Biodiversity, University of Swat, Mingora, Pakistan; 7 State Key Laboratory of Mycology, Institute of Microbiology, Chinese Academy of Sciences, Beijing, 100101 China; 8 Avda. Miranda do Douro 7, 5º G, Aranda de Duero, 09400 Burgos, Spain; 9 Department of Botany, University of Okara, Okara, 56300 Pakistan

**Keywords:** Molecular biology, Plant sciences

## Abstract

In a recent revision of the genus *Agaricus*, *A.* section *Xanthodermatei* was split into two sections *A.* sect. *Hondenses* and *A.* sect. *Xanthodermatei*. Our objectives were to investigate the species diversity of both sections in Pakistan and to give an overview of the major clades. Phylogenetic analyses based on the combined nucLSU, ITS and TEF1 dataset from 35 specimens of both sections revealed three major clades. Analyses based on ITS dataset and 106 specimens, including 33 from Pakistan, reveal eight new species and one new record species. These nine species are described in detail. It is noteworthy that intraspecific variability as well as interspecific variability between closely related species were very low in ITS sequences in many cases. In the case of the two new species *A.* *xanthochromaticus* and *A.* *griseovariegatus*, TEF1 sequence data were much more efficient than ITS to distinguish these species from each other. The other new species are *A.* *atroumbonatus*, *A.* *fumidicolor*, *A.* *macropeplus*, *A.* *parviniveus*, *A.* *swaticus* and *A.* *bambusetorum*. The latter is the only new species of *A.* sect. *Hondenses* in which it is morphologically atypical and also the unique (sub)tropical species. *Agaricus* *gregariomyces* is recorded for the first time in Pakistan. In addition, brief descriptions are provided not only for *A.* *bisporiticus*, *A.* *endoxanthus* and *A.* *punjabensis*, which are reported again in Pakistan, but also for *A.* *californicus*, which is reported for the first time in Spain and outside North America. In total 12 species of both sections were reported in Pakistan and half of them were from subtropical climatic areas, underlining the contribution of the climatic diversity to the high species richness in this country.

## Introduction

*Agaricus* L. (Agaricaceae, Agaricales, Basidiomycota) is a genus of macrofungi containing medicinal and/or nutritional species such as *A.* *subrufescens* Peck and *A.* *bisporus* (J.E. Lange) Imbach, which is the most commonly cultivated mushroom in the world. The genus *Agaricus* currently includes six subgenera, 24 sections, and more than 500 species^1–4^. There is a seventh subgenus *A.* subg. *Conioagaricus* Heinem. containing three sections, *A.* sect. *intermedii* Heinem. (of which type, *A.* *albonudus* Beeli, was combined by Heinemann^5^ himself under *Micropsalliota* Höhn.), *A.* sect. *Pulverotecti* Heinem. and *A.* sect. *Striati* Heinem. Heinemann^6^, which have not been yet studied molecularly in the framework of the new classification system of Zhao et al.^7^. Our taxonomic classification follows the new system proposed by Zhao et al.^7^, a system necessary to accommodate many recently described species and more specifically those from tropical regions.

Over the past six years, many specimens of *Agaricus* have been collected in different ecological and climatic regions in Pakistan. Our study is focused on the samples of this country that are placed in *A.* sect. *Hondenses* R.L. Zhao & L.A. Parra or in *A.* sect. *Xanthodermatei* Singer according to some preliminary investigations. Indeed, all the members of these two sections share the following features, which characterized *A.* sect. *Xanthodermatei*^8,9^ until Zhao et al.^7^ split this section into two sections by introducing *A.* sect. *Hondenses* in the new classification system: (1) positive yellow KOH reaction; (2) negative Schaeffer reaction; (3) more or less pronounced yellow discoloration of the surface especially at the base of the stipe when rubbed, becoming reddish brown over time; and (4) more or less pronounced phenol-like odor. Infrequently, the yellow discoloration and the phenol odor are lacking or not reported. As an example, *A.* *biannulatus* A. Mua, L.A. Parra, Cappelli & Callac was originally described lacking a phenol-like odor according to the remarks of the collectors^10^ but in a subsequent publication^11^ is described with a weak but clear phenol-like odor. Most of the known toxic species of *Agaricus* belong to these sections, in which all species should be considered as potentially poisonous. This mild toxicity causing gastro-intestinal upsets is likely due to the presence of phenol and quinones^12–15^.

Until 2016, *A.* sect. *Xanthodermatei* was included in phylogenetic analyses based on nuc rDNA internal transcribed spacer region (ITS) sequence data^9,16–20^. In these phylogenetic analyses, *A.* sect. *Xanthodermatei* was generally not well-supported and even, in some cases it was not monophyletic. However, three major clades were consistently observed. Indeed, using the maximum likelihood method (ML), Kerrigan et al.^16^ noted that “*A*. sect. *Xanthodermatei* was divided into three ‘subsectional–level’ clades”. One clade comprised species currently placed in *A.* sect. *Hondenses*, another clade included only *A.* *pseudopratensis* (Bohus) Wasser, and the remaining clade was considered as the ‘core’ *Xanthodermatei* group. Thongklang et al.^18^ named these clades Xan I, Xan II and Xan III, respectively. Kerrigan^9^ proposed *A.* subsect. *Hondenses* Kerrigan for the clade that Thongklang et al.^18^ named Xan I. However, earlier that year, Zhao et al.^7^ ranked the clade Xan I as a section not only because its mean stem age (19.35 Ma) was earlier than 18 Ma like those of the other sections of the genus but also because the *A*. sect. *Xanthodermatei* appeared polyphyletic in the phylogenetic analyses of this study. Therefore, in the new classification of Zhao et al.^7^ this section was split into two sections: *A.* sect. *Hondenses* (clade Xan I) and *A.* sect. *Xanthodermatei* (clade grouping clades Xan II and Xan III). Because specimens of these two sections share certain characteristics, it is preferable to study them at the same time despite it remains uncertain that they diverged from a common ancestor. The species diversity in these two sections is well known in Europe^21^ and in North America^9^ and has been more recently investigated in Thailand^18^, China^22^, Iran^20^ and Caribbean^4^.

Our main objectives were to inventory the species diversity in Pakistan and to give an overview of the major clades in these two sections. As parts of the ongoing study on genus *Agaricus* in Pakistan, a first study was focused on *A.* sect. *Brunneopicti* Heinem.^23^, while this second study is focused on *A.* sect. *Hondenses* and *A.* sect. *Xanthodermatei*. Samples collected in Pakistan and included in this study belong to 12 species of these two sections as follows: three species, *A.* *bisporiticus* Nawaz, Callac, Thongklang & Khalid, *A.* *punjabensis* T. Qasim, A. Ashraf & A.N. Khalid, and *A.* *endoxanthus* Berk. & Broome were previously reported from Pakistan by Thongklang et al.^18^, Chen et al.^19^, and Ahmad et al.^24^, respectively; one species, *A.* *gregariomyces* J.L. Zhou & R.L. Zhao, is a new record for Pakistan; the eight remaining taxa are new species, which are described in detail here and included in multi-gene phylogenetic analyses. The 12 species found in Pakistan represent an important contribution to the knowledge of these sections since they are distributed in clade Xan I (*A.* sect. *Hondenses*) as well as in the two clades Xan II and Xan III in *A.* sect. *Xanthodermatei*. In addition, since we reported very closely related species, we examined variable characters among the ITS barcode sequences and we found that both intraspecific and interspecific variabilities were often unexpectedly low for the genus *Agaricus*.

## Materials and methods

### Collected specimens and collection sites in Pakistan

Thirty-five new specimens presumably belonging to *A*. sect. *Xanthodermatei* or *A*. sect. *Hondenses* were selected according to their morphological characteristics. They are indicated in bold type in Table 1. Two of them, LAPAG608 and LAPAM110, were collected in Spain and in Dominican Republic, respectively. These two specimens are deposited in the private herbarium of one of the authors (L. A. Parra). The 33 remaining specimens were collected in different ecological regions of Pakistan during monsoon seasons of 2013–2017. The specimens, including types of the new species, were deposited in LAH (Herbarium, Department of Botany, University of the Punjab, Lahore, Pakistan).

**Table 1:** Taxa, specimens, and GenBank accession numbers of DNA sequences used in phylogenetic analyses.

Major clades	Species^a^	Distribution^b^	Climate^c^	Specimen	GenBank accession numbers		
					ITS	LSU^d^	*Tef1*^*d*^
Xan III C within	***A.*** ***griseovariegatus***	Asia/Pa	TE	**KH167**	**MK101038**	**MK100284**	**MK169406**
***Agaricus*** **subsect**				**BG37**	**KY741891**	**MK100285**	**MK169407**
***Xanthodermatei***				**SA850**	**MG669250**		
				**SA194**	**MG669249**		
				**SA232**	**MK101037**		
	***A.*** ***xanthochromaticus***	Asia/Pa	TE	**KH262**	**MK101034**	**MK100282**	**MK169404**
				**KH270**	**MK101035**		
				**KH295**	**MK101036**	**MK100283**	**MK169405**
	*A.* *sinoplacomyces*	Asia /Si/Yu	SU	ZRL2012009	KM657884		
				ZRL2012008	KM657883	KR006620	KR006648
				ZRL2012028	KM657886		
				ZRL2012027	KM657885		
				ZRLAG2101	KM657887	KR006617	KR006649
	***A.*** ***macropeplus***	Asia/Pa	TE	**MW1501**	**MK141002**	**MK100286**	**MK169408**
				**MW1544**	**MK141003**		
	*A.* sp.			ADK4396	KU041658		
	*A.* *volvatulus*	Africa	TR	LAPAF5	KU041657		
	*A.* sp*.*			NTF58	JF514527		
	*A.* sp*.*			F2767	JF727848		
	*A.* *melanocarpus*	Asia/Yu	SU	ZRL2011037	KM657881		
	*A.* *karstomyces*	Asia/Yu	SU	ZRL2011048	KM657899	KR006632	KR006655
	*A.* *placomyces*	NAm	TE	RWK1959	DQ182525		
	*A.* *berryessae*	NAm	TE	SFSUF-020931	KJ609482		
	*A.* cf. *approximans*	NAm	TE	RWK2064	KJ609480		
	*A.* *moelleri*	Asia/Ir, Eur	TE	LAPAM27	KU041656		
	*A.* *xanthodermus*	Asia/Ir, Eur, NAm	TE	LAPAG387	KM657923	KR006609	KR006638
	*A.* *malangelus*	Asia/Ti, NAm	TE	RWK1971	DQ182522		
				ZRL2012628	KM657892	KR006626	KR006655
	*A.* *menieri*	Eur	TE	CA162	DQ185567		
	*A.* *moelleroides*	Eur	TE	CA215	DQ185559		
	*A.* sp*.*			ZRL2012616	KM657896	KR006630	KR006660
	*A.* *tibetensis*	Asia/Ti	TE	ZRL2012585	KM657895	KR006633	KR006658
	*A.* *deardorffensis*	NAm	TE	ecv4226	KJ609493		
	*A.* *leptocaulis*	NAm	TE	TNF12802	KJ609503		
	***A.*** ***fumidicolor***	Asia/Pa	SU	**CM100**	**KY741306**		
				**CM17**	**KY751305**	N/A^d^	N/A
	*A.* *punjabensis*	Asia/Pa	SU	**CM200**	**MH997901**	**MK100287**	**MK169409**
				**PU265**	**MH997903**		
				A4	KT985909		
				A5	KT985908		
				**L11**	**MH997902**		
	*A.* *endoxanthus*	Cosmopolitan/Pa	SU-TR	ZRL3095	JF691554		
				**J8**	**MK101039**		
				LD2012183	KU041654		
				LAPAM47	KU041655		
				LAPAG598	KU041653		
	*A.* *xanthosarcus*	Africa	TR	Goossens5415	JF514523		
	***A.*** ***atroumbonatus***	Asia/Pa	TE	**MM1637**	**MH997905**	**MK100290**	**MK169412**
				**MM26**	**MH997904**		
	*A.* *daliensis*	Asia/Yu	hot TE	SHY2011071706	KM657877	KR006615	KR006643
	*A.* *atrodiscus*	Asia/Th	TR	LD2012185	KT284912	KT951473	KT951653
	*A.* *iodosmus*	Asia/Ir, Eur, NAm	hot TE	LAPAG245	DQ182518		
	*A.* *tollocanensis*	NAm	TE	CA235	AY703913		
	*A.* *pocillator*	NAm	TE	DUKEJ173	U85308		
	*A.* sp.			F2715	JF727847		
	*A.* sp.			LAPAM61	MF511145		
	*A.* *candussoi*	Car	TR	LAPAM62	MF511146	N/A	N/A
	*A.* sp.			LAPAM20	MF511119		
Xan III B within	***A.*** ***parviniveus***	Asia /Pa	SU	**L20**	**MK101027**		
***Agaricus*** **subsect**				**PU257**	**MK101029**		
***Xanthodermatei***				**PU248**	**MK101028**		
				**PU318**	**MH997906**		
				**NWL314**	**MK007251**		
				**L19**	**MK101026**		
				**TTS42**	**MK101031**		
				**B40**	**MK101030**	**MK100289**	**MK169411**
	***A.*** ***swaticus***	Asia/Pa	TE	**SJ53**	**KY741894**		
				**SA111**	**KY741896**		
				**SJ60**	**KY741895**	**KY741902**	N/A
	*A.* *langensis*	Asia/Ti	TE	ZRL20152282	MG763129	MG765264	MG765266
	*A.* sp.			ZRL2012629	KM657890	KR006627	KR006656
	*A.* *parvitigrinus*	Eur	TE	CA158	AY899267		
	*A.* *californicus*	NAm	TE	RWK1914	DQ182509		
		Eur	TE	**LAPAG608**	**MK215826**		
	*A.* *laskibarii*	Eur	TE	LAPAG115	AY943975		
	*A.* *xanthodermulus*	Eur	TE	CA160	AY899273		
	*A.* *caribaeus*	Car	TR	LAPAM41	MF511130		
	*A.* sp.			LAPAM46	MF511134		
	*A.* *arizonicus*	NAm	TE	FRMC1255	KJ609481		
Xan III A within	*A.* *flavidodiscus*	Car	TR	LAPAM17	MF511116	MF511152	MF511153
***Agaricus*** **subsect**							
***Xanthodermatei***							
Xan II	*A.* *murinocephalus*	Asia/Th	TR	ZRL3044	JF691555		
***Agaricus*** **subsect**	*A.* *fuscopunctatus*	Asia/Th	TR	LD2012115	KJ575612		
***Paradoxi***	*A.* *exilissimus*	Asia/Th	TR	MFLU12 0894	KT284910		
	*A.* sp*.*			ZRLWXH3092	KM657891	KR006619	KR006646
	*A.* sp.			2E6	AB973764		
	*A.* *tytthocarpus*	Asia/Fu	SU	ZRLWXH3077	KM657889	KR006618	KR006645
	*A.* *bisporiticus*	Asia/Pa/Th	SU-TR	LD2012111	KJ575611	KT951507	KT951650
				**PU252**	**MK101033**		
	*A.* *tephrolepidus*	Car	TR	LAPAM18	MF511117		
	*A.* *gregariomyces*	Asia/Pa/Ti	TE	**KH72**	**MK101032**		
				ZRL2012624	KM657880	KR006625	KR006653
	*A.* *buckmacadooi*	NAm	TE	SFSU B49	KJ609484		
	*A.* *kriegeri*	NAm	TE	SFSU2079	KJ609500		
	*A.* *memnonius*	Asia/Si	hot TE	ZRL20151118	MG763128	MG765263	MG765265
	*A.* *microvolvatulus*	Africa, Asia/Th	TR	LD201271	KJ575614	KT951508	KT951651
	*A.* sp*.*			**LAPAM110**	**MK508864**		
	*A.* *pseudopratensis*	Eur	TE	LAPAG259	DQ182527	**MK123324**	**MK169403**
	*A.* *brunneogracilis*	Asia/Th	TR	ZRL258	KM657876	KR006628	KR006657
Xan I	*A.* *subrufescentoides*	NAm	TE	BM09D	KJ609508		
*Agaricus* sect	*A.* *pusillobulbosus*	Asia/Ti	TE	ZRL2012627	KM567888	N/A	KR006654
*Hondenses*	*A.* *hondensis*	NAm	TE	RWK1938	DQ182513		
	*A.* *freirei*	Eur	TE	CA187	DQ185554		
	*A.* *grandiomyces*	Asia/Ti	TE	ZRL2012611	KM657879	KR006624	KR006652
	***A.*** ***bambusetorum***	Asia/Pa	SU	**CM242**	**MH923515**	**MK100288**	**MK169410**
				**NWL322**	**MH923516**		
	*A.* *biannulatus*	Eur	TE	LAPAG611	JF896229	N/A	**MK291951**
	*A.* *phaeolepidotus*	Asia/Ir, Eur	TE	CA217	DQ185552		
Outgroup	*A.* *langei* [*A.* sect. *Agaricus*]			LAPAG141	JF797181		
	*A.* *campestris* [*A*. sect. *Agaricus*]			LAPAG370	KM657927	KR006607	KR006636
	*A.* *variicystis* [*A*. sect. *Crassispori*]			LD201234	KT951339	KT951517	KT951562
	*A.* *trisulphuratus* [*A*. sect. *Trisulphurati*]			LAPAF7	KM657924	KR006605	KR006634
	*A.* *cupressicola* [*A*. sect. *Bivelares*]			LAPAG889	KT951334	KT951465	KT951649
	*A.* *bisporus* [*A*. sect. *Bivelares*]			LAPAG446	KM657926	KR006610	KR006639

New taxa, new specimens and new accession GenBank numbers are in bold type.

^a^Species are grouped by clade. Within each clade, they are arranged in the order in which they appear in the ITS phylogenetic tree represented in Fig. 2.

^b^Abbreviations: Car (Caribbean), Eur (Europe), NAm (North America); Asian countries: Ir (Iran), Pa (Pakistan), Th (Thailand); Chinese provinces: Fu (Fujian), Si (Sichuan), Ti (Tibet), Yu (Yunnan).

^c^Abbreviations: SU (subtropical), TR (tropical), TE (temperate or other non-tropical climates).

^d^LSU and Tef1 GenBank accession numbers are indicated only for 40 samples used in multi-gene analysis; N/A means not available.

Among the 33 specimens collected in Pakistan, 17 were collected in subtropical climate areas and 16 in temperate climate areas. The subtropical areas include Changa Manga forest, Kasur district and Lahore. The temperate areas include the moist temperate regions of Bagh, Shangla and Swat districts, and the dry temperate regions of Kalam and Mashkun in Swat district.

Lahore is the capital of Punjab province lying at about 217 m a.s.l. The monthly mean temperature ranges between 10 and 38° C during the year in Lahore. (http://rmcpunjab.pmd.gov.pk/rmc-HistoricalMaxTemp.php). Changa Manga forest lies in the southeast of Lahore district with an elevation of 214 m a.s.l. and localized between 31º N and 73º E. The monthly mean temperature ranges from 5.9 to 39.6 ºC^25^ and annual rainfall recorded is about 650 mm, mostly received during the monsoon season^26^.

The Bagh district lies between 73° and 75° E and 33°–36° N in the western Himalayas. Monthly mean temperature in the area varies from 5 to 32 °C and the lowest temperature was − 2 °C, recorded in December. This region lies in monsoon range and the annual precipitation is about 370 mm to slightly more than 390 mm^27^. The Swat district lies between 34° and 35° N and 72°–74° E in the Khyber Pakhtunkhwa Province of Pakistan. This region occupies the nexus of three mountain ranges of the Himalayas, Hindu Kush and Karakoram^28^. Kalam is a dry temperate region in Swat, with an elevation of 2060–2065 m a.s.l. dominated by *Cedrus* *deodara*, *Quercus* *oblongata* forests along with *Pinus* species^29,30^. The Shangla district has an average altitude of 2000–3500 m, and lies between 34° and 33° N and 72°–73° E. The weather of the area is mild in summer and cold in winter. The annual rainfall is approximately 1415.9 mm^31^.

### Morphological observations

Specimens were photographed at collection sites. Collected basidiomata were vouchered and dried by a fan heater. Macroscopic characters were noted such as color, size and shape of pileus, stipe and lamellae, generally following Largent^32^ and Chen et al.^33^. The fresh sporocarp characters were carefully noted in the field viz. discoloration upon bruising, odor and Schaeffer’s reaction. Color notations were indicated from the Munsell’s Soil Color Charts^34^.

For microscopic observations, slides were prepared in 5% aqueous KOH (w/v) and then stained in 1% aqueous Congo red solution (w/v). Microscopic features like, size and shape of basidiospores, basidia, cheilocystidia, pileipellis, stipitipellis and hyphae of lower surface of the annulus were measured using a light microscope (MX4300H, Meiji Techo Co., Ltd., Japan). Measurements of anatomical features (basidiospores, basidia and cheilocystidia) were presented based on at least 30 measurements. The dimensions of basidiospores are given in the form of (a) b–c (d) × (e) f–g (h), [avX, Qm, n = i × j] where b–c and f–g include the spore length and width respectively between the 5th percentile and the 95th percentile, (a) and (d) the extreme values of spore lengths recorded, (e) and (h) the extreme values of spores width recorded, avX the mean of length by width ± SD (standard deviation), Qm the mean of Q coefficient (length/width ratio), n is the total number (i) of spores measured of each collection and (j) number of total collections measured. Measurements of other microscopic structures (basidia, cheilocystidia, pileipellis, stipitipellis and hyphae of lower surface of annulus where the piece of annulus was available in dried samples) include the range between the extreme values measured in length and width. Basidia were measured excluding sterigmata.

### Sampling for phylogenetic analyses and for comparisons between ITS sequences

For phylogenetic analyses, we used sequence data of 107 specimens of 72 species or putative species of *A*. sect. *Xanthodermatei* or *A*. sect. *Hondenses* of which 59 are named species. All 72 species except the eight new species described in this study and *A.* sp./LAPAM110 have been included in previous phylogenetic analyses^4,7,9,10,16–20,35–37^. For the multi-gene analysis, 35 of the 107 specimens were used. They belong to 32 species of which 29 are named species. For *A.* *malangelus* Kerrigan, a representative from China with complete multigene dataset was used. However, we preferred to include the type specimen from North America to represent this species in phylogenetic analyses based on ITS dataset. Consequently, in the latter analyses, we used only 106 of the 107 samples of both sections listed in Table 1. In addition to the 107 specimens mentioned above, six specimens from six species belonging to different sections known to be closely related to *A*. sect. *Xanthodermatei* and *A*. sect. *Hondenses*, were used as outgroup. Taxon names (sections, subsections and species), specimens, and all GenBank accession numbers are indicated in Table 1.

To compare the variability within species and between very closely related species, ITS sequences of 28 specimens were aligned. Their GenBank accession numbers are in Table 1 except for the nine following specimens which were not used in the phylogenetic analyses to avoid redundancy: four of *A.* *deardorffensis* Kerrigan (BM09E/KJ609492, RWK2003/KJ609495, RWK2004/KJ609496 and RWK2028/ J609497), one of *A.* *leptocaulis* Kerrigan (MO24015/KJ609502), two of *A.* *tibetensis* J.L. Zhou & R.L. Zhao (ZRL2012580/KR006604 and ZRL2012617/KM657897), and two of unidentified specimens (ZRL2012474/KM657893 and ZRL2012582/KM657894). In addition, we compared 30 sequences deposited as *A.* *xanthodermus* Genev. in GenBank. After discarding misidentified samples it remained 18 samples with the following GenBank accession numbers: AJ418776, AY484689, AY899271, AY899272, DQ182529, DQ182534, DQ185563, DQ185564, DQ185565, KJ609510, KM657923, KM657925, KT824788, KT824789, KX098651, KX098652, KX098653, MH752460.

ITS sequence data of ZRL2012474, ZRL2012582, and ZRL2012616 were used in Zhou et al.^22^ where these three samples were neither described nor identified. They are incorrectly identified as *A.* *deardorffensis* in GenBank (KM657893, KM657894, and KM657896 respectively). In addition, although the ITS sequences of ZRL2012474 and ZRL2012582 are highly similar, their translation elongation factor 1-α gene (TEF1) sequences available in GenBank differ at 13 positions. We included these two samples in our Table of comparison of ITS sequences but until these data would be confirmed we prefer not to include them in our phylogenetic analyses.

### DNA extraction, PCR and sequencing

DNA from dried specimens was extracted using a modified 2% CTAB protocol as described by Zhao et al. (2011). PCR amplification of the nuc rDNA internal transcribed spacer region (ITS1-5.8S-ITS2 = ITS), nuc rDNA large subunit fragment (LSU) and translation elongation factor 1-α gene fragment (TEF1) was performed using the combination of primers ITS1F/ITS4^38,39^, LROR/LR5^40^ and EF1-983F/EF1-1567R^41^, respectively. The PCR products were sequenced on both strands at BGI (Beijing Genomics Institute), Hong Kong, TsingKe, China and Genewiz, UK. All the sequences generated in this study have been submitted to GenBank.

### Sequence alignment and phylogenetic analyses

Sequences were aligned for each region independently, using T-coffee version 8.99^42^ then the alignment was adjusted manually in Bioedit version 7.2.0. The maximum likelihood analyses were performed on the CIPRES PORTAL v. 3.1.^43^. The phylogenetic trees were inferred for each alignment using RAXML–HPC2 v 8.1.11^44^. Rapid bootstrapping was performed with 1000 bootstrap iterations. Significant support was considered to be BS ≥ 50. There was no significant incongruence between the datasets, so the ITS, LSU, and TEF1 sequences were concatenated in BioEdit for subsequent phylogenetic analyses. The combined dataset was partitioned into ITS, LSU, TEF1 intron and TEF1 coding sites. The best substitution model for each partition was inferred with the program MrModeltest version 2.2^45^: SYM + G for all partitioned regions. Bayesian Inference (BI) analysis was performed with MrBayes v. 3.1.2^46^. Six Markov chains were run for one million generations and sampled every 100th generation. Burn–in was determined in TRACER v. 1.6^47^ from the likelihood trace plots and subsequently discarded. The resulted trees were displayed in FigTree version 1.4.2 (http://tree.bio.ed.ac.uk/software/figtree/). The final alignments of ITS and multi-gene dataset were deposited in TreeBASE (ID = 26651).

### Taxon-specific ITS markers

Markers characterizing species, clades, sub-sections or sections were identified in the alignment of 106 ITS sequences of specimens belonging to *A*. sect. *Xanthodermatei* or *A*. sect. *Hondenses*. These markers are indicated by uppercase letters and are given along with flanking sequences. IUPAC codes, such as Y, do not indicate ambiguity (C or T) but heteromorphism (C and T) likely reflecting allelic polymorphism in the heterokaryotic (*n* + *n*) basidiomata. Insertions or deletions (indel) are indicated in square brackets. The places of the markers are numbered according to their position either in the 5’-3’ ITS1–5.8S–ITS2 DNA sequence (beginning by tygaatt) of the characterized species, or in the alignment deposited in TreeBASE for the characterization of clades or higher taxa.

## Results

### Phylogenetic analyses

For phylogenetic analyses, 57 sequences were newly generated (35 ITS, 11 LSU, and 11 TEF1) from 37 specimens. Among these, 33 specimens belong to 12 species from Pakistan including eight new species described in this study, one specimen was from Dominican Republic (LAPAM110 as *A.* sp.), and three from Europe: LAPAG608 (*A.* *californicus*), LAPAG611 (*A.* *biannulatus*) and LAPAG259 (*A.* *pseudopratensis*). ITS sequences of the latter two specimens were previously obtained, while the 35 remaining specimens were included for the first time in phylogenetic analyses. All samples with their GenBank accession numbers are listed in Table 1.

The alignment for multi-gene analyses consisted of sequences (ITS/LSU/TEF1) of 40 specimens and 2099 characters. The percentage of missing data was 6% (7/120) due to seven non-obtained sequences. The five samples used as outgroup belong to five different species of four different sections: *A.* sect. *Bivelares* (*A.* *bisporus* and *A.* *cupressicola*), *A*. sect. *Agaricus* (*A.* *campestris*), *A.* sect. *Crassispori* (*A.* *variicystis*) and *A.* sect. *Trisulphurati* (*A.* *trisulphuratus*). The 35 remaining samples belonged to 32 species or putative species of *A.* sect. *Xanthodermatei* or *A.* sect. *Hondenses* (Table 1).

The alignment for ITS dataset consisted of sequences of 108 specimens and 709 characters. Two samples of species belonging to *A*. sect. *Agaricus* were used as outgroup (*A.* *campestris* and *A.* *langei*), while the 106 remaining sequences belonged to 72 species or putative species of *A.* sect. *Xanthodermatei* and *A.* sect. *Hondenses*.

**Figure 1:**
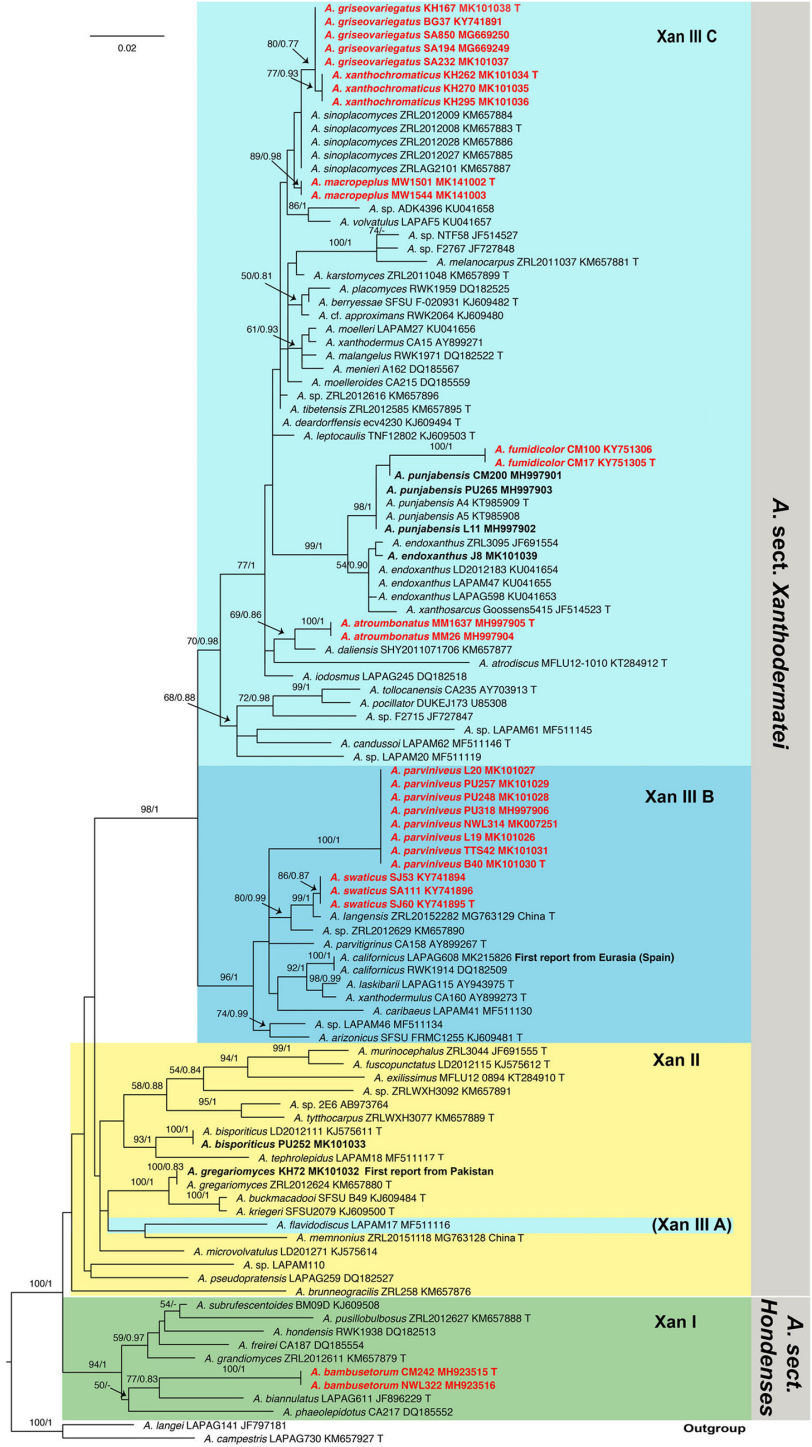
Maximum likelihood tree of species belonging to *A*. sect. *Xanthodermatei* and *A*. sect. *Hondenses* generated from combined LSU, ITS and TEF1 sequence data. Four species of closely related sections (in *A.* subg. *Pseudochitonia*) and *A.* *campestris* (in *A.* subg. *Agaricus*) are used as outgroup. Bootstrap support values and Bayesian posterior probabilities are indicated (BS/PP). New species from Pakistan are in bold red. *T* type specimen.

**Figure 2:**
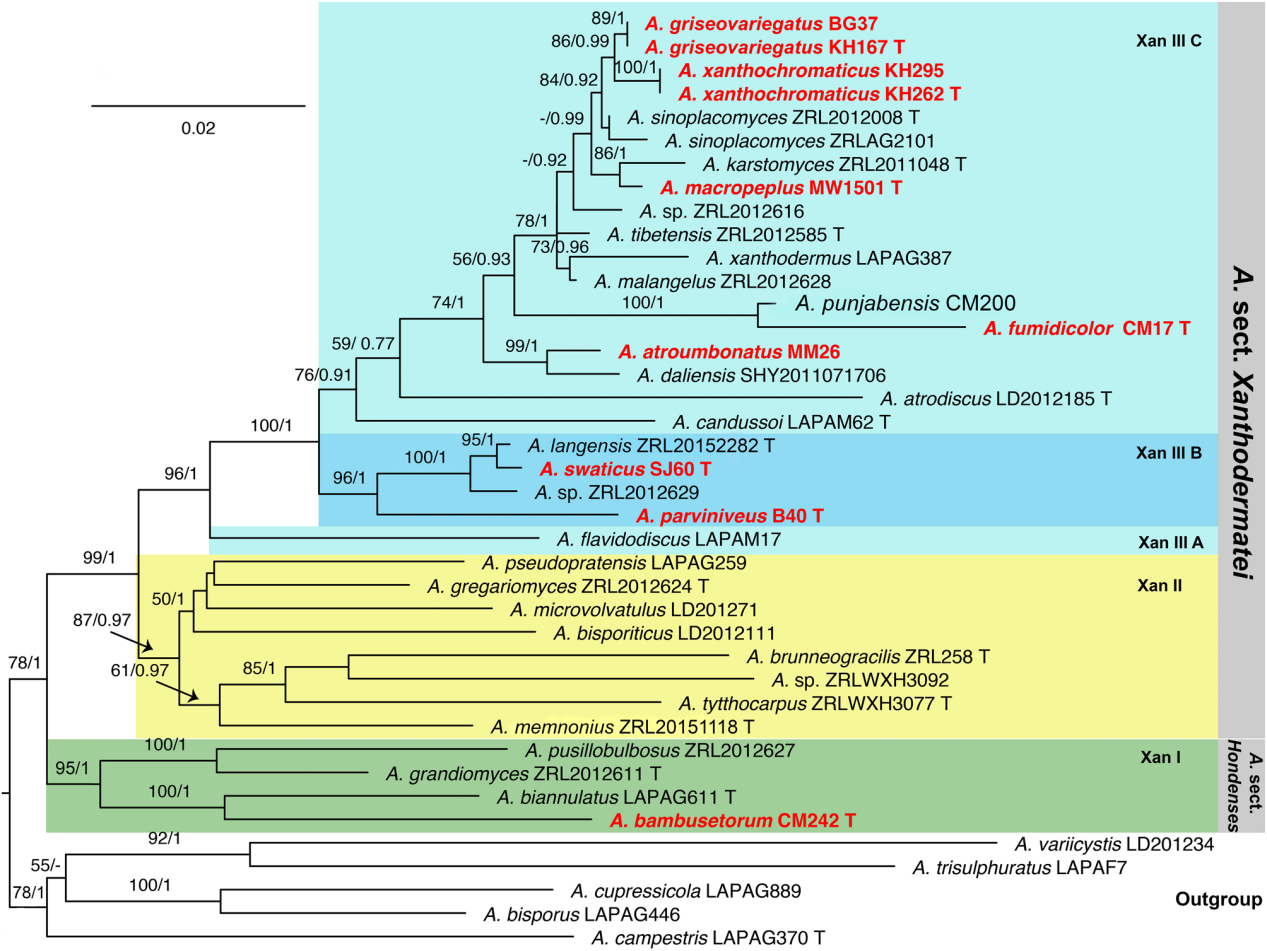
Maximum likelihood tree of species belonging to *A*. sect. *Xanthodermatei* and *A*. sect. *Hondenses* generated from ITS sequence data. *Agaricus* *campestris* and *A.* *langei* are used as outgroup. Bootstrap support values and Bayesian posterior probabilities are indicated (BS/PP). New samples from Pakistan are in bold and new species in bold red. *T* type specimen.

For both multi-gene and ITS analyses, the topologies of the trees using the Maximum likelihood (ML) or the Bayesian inference (BI) methods were highly similar. The bootstrap support values (BS) and Bayesian posterior probabilities (PP) are reported in the ML phylograms of Fig. 1 for multi-gene analyses, and Fig. 2 for ITS analyses.

Despite we completed multi-gene sequence dataset for many new samples and some previous samples it remains many species only having ITS sequence data. For this reason we present first the phylogeny of the sections and major clades based one multi-gene-analyses. The ITS analysis remains useful mainly at species level to circumscribe new taxa and identify their most closely related taxa.

### Phylogeny and molecular characterization of sections and major clades

The major clades Xan I, Xan II and Xan III are those previously proposed by Thongklang et al. (2014). In multi-gene tree (Fig. 1), *A*. sect. *Hondenses*, which corresponds to the clade Xan I, and *A*. sect. *Xanthodermatei* are both monophyletic and statistically well supported (BS/PP = 95/1 and 99/1, respectively). *Agaricus* sect. *Xanthodermatei* is subdivided into two major clades, Xan II and Xan III, which are well supported (BS/PP = 87/0.97 and 96/1, respectively). In addition, Xan III is subdivided into three clades, which are named Xan III A (monospecific), Xan III B (BS/PP = 96/1), and Xan III C (BS/PP = 76/0.91).

Comparing the ITS barcode sequences of 72 species or putative species of clades Xan I, Xan II and Xan III, we found three clade-specific markers. Two markers are indel mutations (insertion or deletion), which are sufficient to characterize the three clades:

cyyvrt[YTHRYSR]gacaat@684–690, this 7-bp insertion characterizes Xan I;

tcagyw[–]tygctg@148, the 1-bp deletion characterizes the clade Xan II;

crgaacCryttgy@590, the nucleotide C characterizes Xan I.

In the current stage of knowledge, these molecular clade-specific markers at ITS positions 148, 590 and 684–690 allow to classify the species in the three clades Xan I, Xan II and Xan III and comfort the existence of these clades. These clades were revealed in multi-gene phylogenetic analyses despite the used algorithms did not take indel in consideration.

### Placement of 33 specimens from Pakistan in the phylogenetic tree

The 72 species or putative species included his study were distributed in sections or major clades as follows: eight in *A.* sect. *Hondenses* (clade Xan I) and 64 in *A.* sect *Xanthodermatei*; 16 in Xan II and 48 in Xan III; 1, 11, and 36 in Xan III A, Xan III B, and Xan III C, respectively.

Six of the 33 specimens newly collected in Pakistan belong to four previously described species of *A.* sect. *Xanthodermatei* in the ITS tree (Fig. 2). Two of the six (KH72 and PU252) are grouped in Xan II. The ITS sequence of the specimen KH72 is identical to that of the type specimen of *A.* *gregariomyces* known from China and is the first report of this species in Pakistan. The ITS sequence of the specimen PU252 is identical to that of the type specimen of *A.* *bisporiticus,* a species already known from Pakistan and Thailand^18^. The four remaining specimens match with two species in clade Xan III C. The sequences of the three specimens PU265, L11 and CM200 are identical or differ at one position from the type specimen of *A.* *punjabensis*, a species previously described from Pakistan. The specimen J8 has a sequence that differs at one position from the sequences of the cosmopolitan species *A.* *endoxanthus*. It is not rare to find one or even more differences between sequences of specimens of species such as *A.* *punjabensis* and *A.* *endoxanthus* that exhibit large genetic variability with numerous heteromorphisms and polymorphic positions^16^. Phylogenetic data support the belonging of these six new specimens to four species previously reported from Pakistan.

The 27 remaining specimens from Pakistan are grouped in eight clades in the ITS tree (Fig. 2), which represent eight newly described species in this study. Five of these clades are well supported (BS > 80 and PP > 0.95). The clade of the new species *A.* *swaticus* is relatively well supported (BS = 86 and PP = 0.87) and sister to *A.* *langensis* M.Q. He & R.L. Zhao, a species recently described from China^35^. We note that despite the ITS sequences of all three specimens of *A.* *swaticus* differ at only two positions from the sequence of *A.* *langensis*, the divergence node between the two species is well supported. The two remaining clades correspond to the two sister species *A.* *griseovariegatus* and *A.* *xanthochromaticus*. Their ITS sequences differ at only one position. Because such a low level of divergence between species is infrequent in the genus *Agaricus*, we included two specimens of each of these two species in the multi-gene analysis. In the multi-gene tree, the clades corresponding to *A.* *griseovariegatus* and *A.* *xanthochromaticus* are well supported and have longer branches due to differences at six additional positions in TEF1 sequences and one in LSU sequences. In addition, the node of divergence between these two species and, more generally, between the eight new species and their closest relative are well supported except for *A.* *macropeplus*. Indeed, the latter is not clearly related to any species in the ITS tree but appears closely related to *A.* *karstomyce*s in multi-gene tree. *Agaricus* *bambusetorum* takes place in *A*. sect. *Hondenses*, while the seven remaining new species are in *A*. sect. *Xanthodermatei*, either in clade Xan III B (*A*. *swaticus* and *A.* *parviniveus*) or in clade Xan III C (*A.* *atroumbonatus*, *A.* *fumidicolor*, *A.* *griseovariegatus,* *A.* *macropeplus* and *A.* *xanthochromaticus*).

### Inter- and intra-specific variability

Among ITS sequences of 27 specimens belonging to the eight new species described in this study, and also among LSU and TEF1 sequences of four specimens belonging to two of these species, we noted a quasi-absence of intraspecific variability. The only exception is *A.* *parviniveus*, in which four heteromorphisms were found in samples B40 and PU257*,* at three and one positions of their ITS sequences, respectively. In addition, we noted a low level of genetic divergence between closely related species such as *A.* *griseovariegatu*s and *A.* *xanthochromaticus*, which differ at only one position of their ITS sequences. Based on these preliminary observations of a low variability among ITS sequences of our new specimens, we extended this investigation to other species of *A.* sect. *Xanthodermatei*. On the one hand, we examined the intraspecific variability among numerous available ITS sequences of *A.* *xanthodermus*, the type species of *A*. sect. *Xanthodermatei*. On the other hand, we examined the inter- and intra-specific variability of the ITS region and of the spore size in a group of ten closely related named or putative species in the clade Xan III C, which includes three of our new species (*A.* *griseovariegatu*s, *A.* *macropeplus* and *A.* *xanthochromaticus*).

To know if the high genetic homogeneity observed in our new species also applies to *A.* *xanthodermus*, which is probably the most represented species of the section in GenBank, we retrieved 30 ITS sequences having been deposited as *A.* *xanthodermus*. We first discarded 12 sequences of misidentified specimens which belong to more or less closely related species such as for example three sequences of specimens from China (JX434654, EU326208 and EU273511), which are 100% identical to sequences of *A.* *sinoplacomyces* Callac & R.L. Zhao and differ at 8 positions from *A.* *xanthodermus*. A second example consists of two samples from Ethiopia (KP229414 and KP229415; Sitotaw et al.^48^), which are closely related to the tropical species *A.* *volvatulus* Heinem. & Gooss.-Font. (1–3 differences) described from Africa and which differ at 11 positions from *A.* *xanthodermus*. We found that the 18 remaining sequences are 100% identical except VM101/KT824788, which differs at one position (T insertion). These 18 samples are from Europe, Iran, USA or Canada. Therefore, based on ITS data, *A.* *xanthodermus* is a highly homogeneous species although it is distributed in different continents. We also note that various species of the section are frequently misidentified as *A.* *xanthodermus*.

The alignment of 28 ITS sequences belonging to ten closely related species revealed 18 variable nucleotide sites. Molecular characters at these 18 positions are indicated in Table 2. In this table, the 28 sequences are arranged as far as possible in the order in which they would have evolved from the most consensual sequence of *A.* *deardorffensis* and following the most parsimonious sequence of events. This order agrees with the order in which these species appear in the ITS tree where the position of *A.* *deardorffensis* is ancestral-like relatively to this group of species. A similar order is observed for the six species of this group that are represented in the multi-gene tree. In this group of species, the intraspecific variability is quasi-absent except in *A.* *leptocaulis* and except two heteromorphisms found in a sample of *A.* *tibetensis* and in a sample of *A.* *placomyces* Peck. In addition, the number of differences between closest related species is very low since it varies from 1 to 3.

**Table 2:** Comparison of 15 ITS sequences of three new species (*A.* *macropeplus*, *A.* *griseovariegatus* and *A.* *xanthochromaticus*) with 13 sequences of closely related taxa.

Specimen^a^			Variable position^b^																	Species	Country
	201	447	242	260	496	127	39	513	494	37	207	518		503	73	500	20	91	259		
TNF 12802T	G	A	**Y**	**R**	**R**	C	A	G	C	**Y**	C	C		*T*	–	*G*	*C*	T	T	*A.* *leptocaulis*	USA
MO 24015	G	A	C	A	G	C	A	G	C	C	C	C		*T*	–	*G*	T	T	T		Canada
ECV4226 T	G	A	C	A	G	C	A	G	C	C	C	C		C	–	A	T	T	T	*A.* *deardorffensis*	USA
RWK 2028	G	A	C	A	G	C	A	G	C	C	C	C		C	–	A	T	T	T		
RWK 2003	G	A	C	A	G	C	A	G	C	C	C	C		C	–	A	T	T	T		
RWK 2004	G	A	C	A	G	C	A	G	C	C	C	C		C	–	A	T	T	T		
BM-09E	G	A	C	A	G	C	A	G	C	C	C	C		C	–	A	T	T	T		
ZRL2012585 T	G	A	C	A	G	C	A	G	C	C	C	*A*		C	–	A	T	T	T	*A.* *tibetensis*	China
ZRL2012580	G	A	C	A	G	C	A	G	C	C	C	*A*		C	–	A	T	T	T		
ZRL2012617	G	**R**	C	A	G	C	A	G	C	C	C	*A*		C	–	A	T	T	T		
ZRL2012616	G	A	C	A	G	C	A	G	C	C	C	*A*		C	–	A	T	*G*	T	*A.* *sp.*	China
ZRL2012474	G	A	C	A	G	C	A	G	C	C	*T*	*A*		C	–	A	T	T	*–*	*A.* *sp.*	China
ZRL2012582	G	A	C	A	G	C	A	G	C	C	*T*	*A*		C	–	A	T	T	T	*A.* *sp.*	China
MW1501 T	G	A	C	A	G	C	A	G	C	*T*	*T*	*A*		*T*	*T*	A	T	T	T	*A.* *macropeplus*	Pakistan
MW1544	G	A	C	A	G	C	A	G	C	*T*	*T*	*A*		*T*	*T*	A	T	T	T		
ZRL2012008 T	G	A	C	A	G	C	A	G	*T*	*T*	*T*	*A*		C	–	A	T	T	T	*A.* *sinoplacomyces*	China
ZRL2012027	G	A	C	A	G	C	A	G	*T*	*T*	*T*	*A*		C	–	A	T	T	T		
ZRL2012009	G	A	C	A	G	C	A	G	*T*	*T*	*T*	*A*		C	–	A	T	T	T		
ZRL2012028	G	A	C	A	G	C	A	G	*T*	*T*	*T*	*A*		C	–	A	T	T	T		
ZRLAG2101	**R**	A	C	A	G	C	A	G	*T*	*T*	*T*	*A*		C	–	A	T	T	T		
KH167 T	G	A	C	A	G	C	*G*	*A*	*T*	*T*	*T*	*A*		C	–	A	T	T	T	*A.* *griseovariegatus*	Pakistan
SA232	G	A	C	A	G	C	*G*	*A*	*T*	*T*	*T*	*A*		C	–	A	T	T	T		
SA850	G	A	C	A	G	C	*G*	*A*	*T*	*T*	*T*	*A*		C	–	A	T	T	T		
BG37	G	A	C	A	G	C	*G*	*A*	*T*	*T*	*T*	*A*		C	–	A	T	T	T		
SA194	G	A	C	A	G	C	*G*	*A*	*T*	*T*	*T*	*A*		C	–	A	T	T	T		
KH262 T	G	A	C	A	G	*T*	*G*	*A*	*T*	*T*	*T*	*A*		C	–	A	T	T	T	*A.* *xanthochromaticus*	Pakistan
KH270	G	A	C	A	G	*T*	*G*	*A*	*T*	*T*	*T*	*A*		C	–	A	T	T	T		
KH295	G	A	C	A	G	*T*	*G*	*A*	*T*	*T*	*T*	*A*		C	–	A	T	T	T		

Nucleotides differing from the most consensual sequence (*A.* *deardorffensis*) are highlighted in italics. Heteromorphisms reflecting intraspecific polymorphisms are highlighted in bold. Specimens and variable positions are arranged in order to facilitate comparison between species.

^a^Genbank accession numbers of 19 specimens used in the phylogenetic analysis are given in the ITS tree of Fig. 1 and in Table 1. Those of the nine remaining samples are given in Material and Methods.

^b^Variable characters are numbered according to their positions in an alignment including the 28 ITS sequences used in the table.

**Figure 3:**
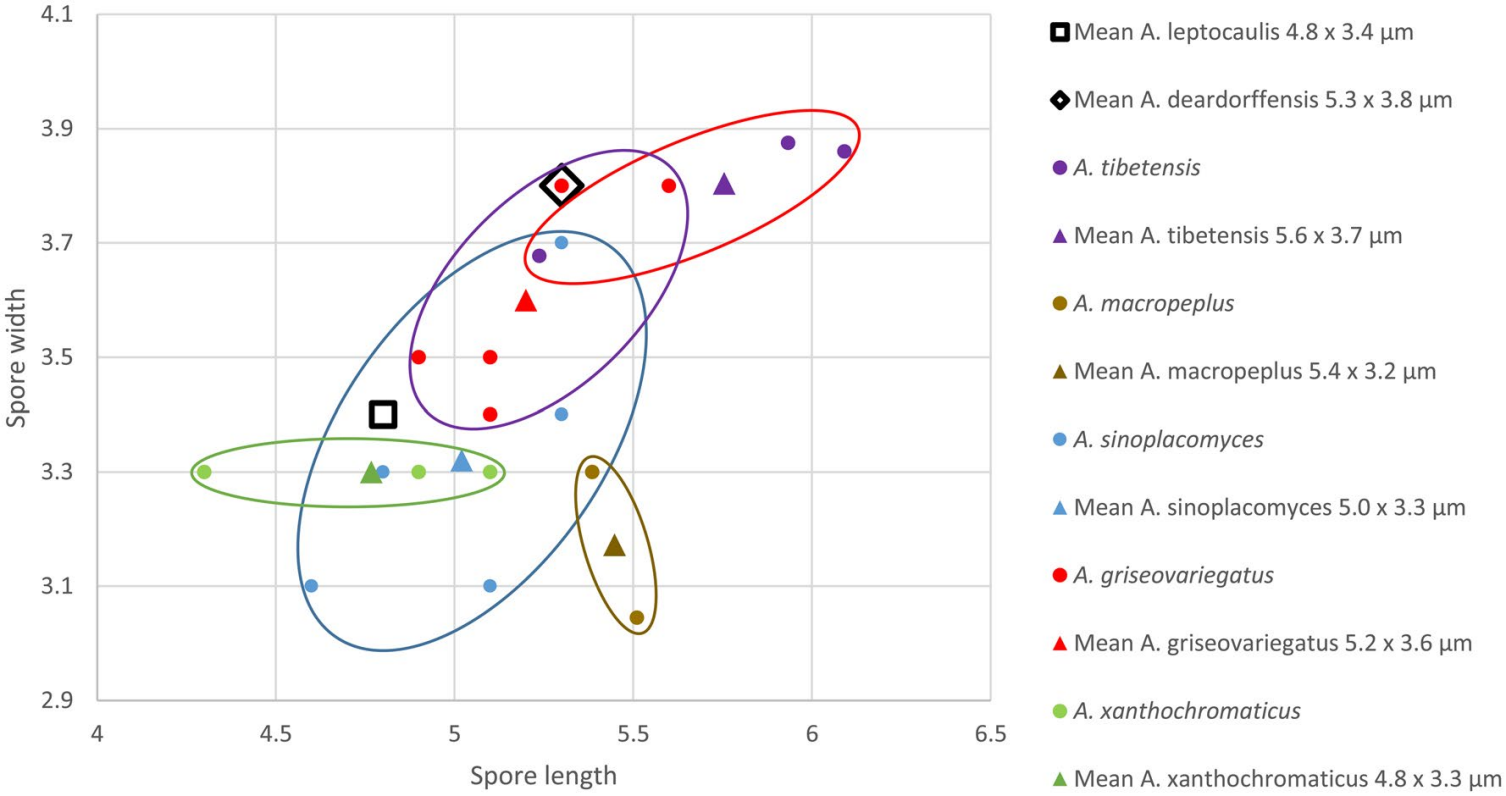
Graphical of spore size comparisons between seven closely related species including the three new species *A*. *griseovariegatus*, *A.* *macropeplus*, and *A.* *xanthochromaticus*.

The spore sizes of the seven named species of this group are compared in the Fig. 3. For the five species known from Asia, the spore sizes were measured on the same 18 specimens that were used in the Table of comparison of ITS sequences, while only mean spore sizes reported in Kerrigan (2016) are represented for the two species from North America. Except for *A.* *tibetensis*, of which the mean spore size appears higher than those of the other species (5.75 × 3.80 µm) but to a lesser extent than the value (6.8 × 4.2 µm) reported by Zhou et al.^22^, spore sizes are not useful to reliably distinguish the species of this group from each other.

## Taxonomy

### Detailed descriptions of one newly reported species and eight new species from Pakistan

***Agaricus*** **subg*.*** ***Pseudochitonia*** Konrad & Maubl., Icon. Select. Fung. 6, fasc. 3: 61. 1927

MycoBank**:** MB625878

*Type:* ***Agaricus*** ***pequinii*** (Boud.) Konrad & Maubl., Icon. Select. Fung. 6(3): 61. 1927

***Agaricus*** **sect.** ***Hondenses*** R.L. Zhao & L.A. Parra, Fungal Diversity 78(1): 272. 2016

MycoBank: MB570227

Type: ***Agaricus*** ***hondensis*** Murrill, Mycologia 4: 296. 1912

**Figure 4:**
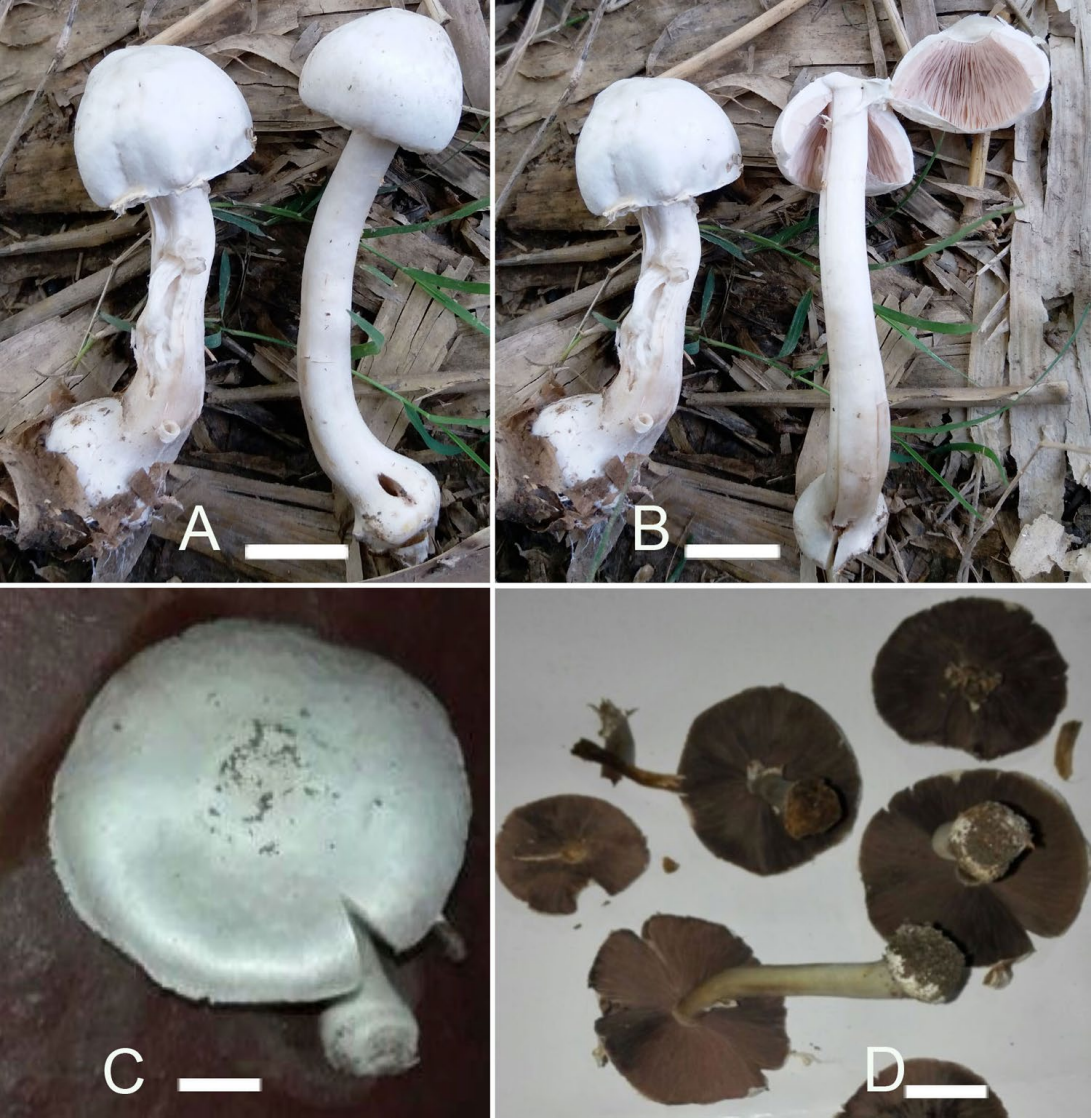
Macromorphological characters of *A.* *bambusetorum.* **(A–D)** basidiomata in the field. **(A,B)**  CM242 (**Holotype**), **(C,D)** = NWL322. Bars = 1 cm. Photographed by Dr. Hira Bashir.

**Figure 5:**
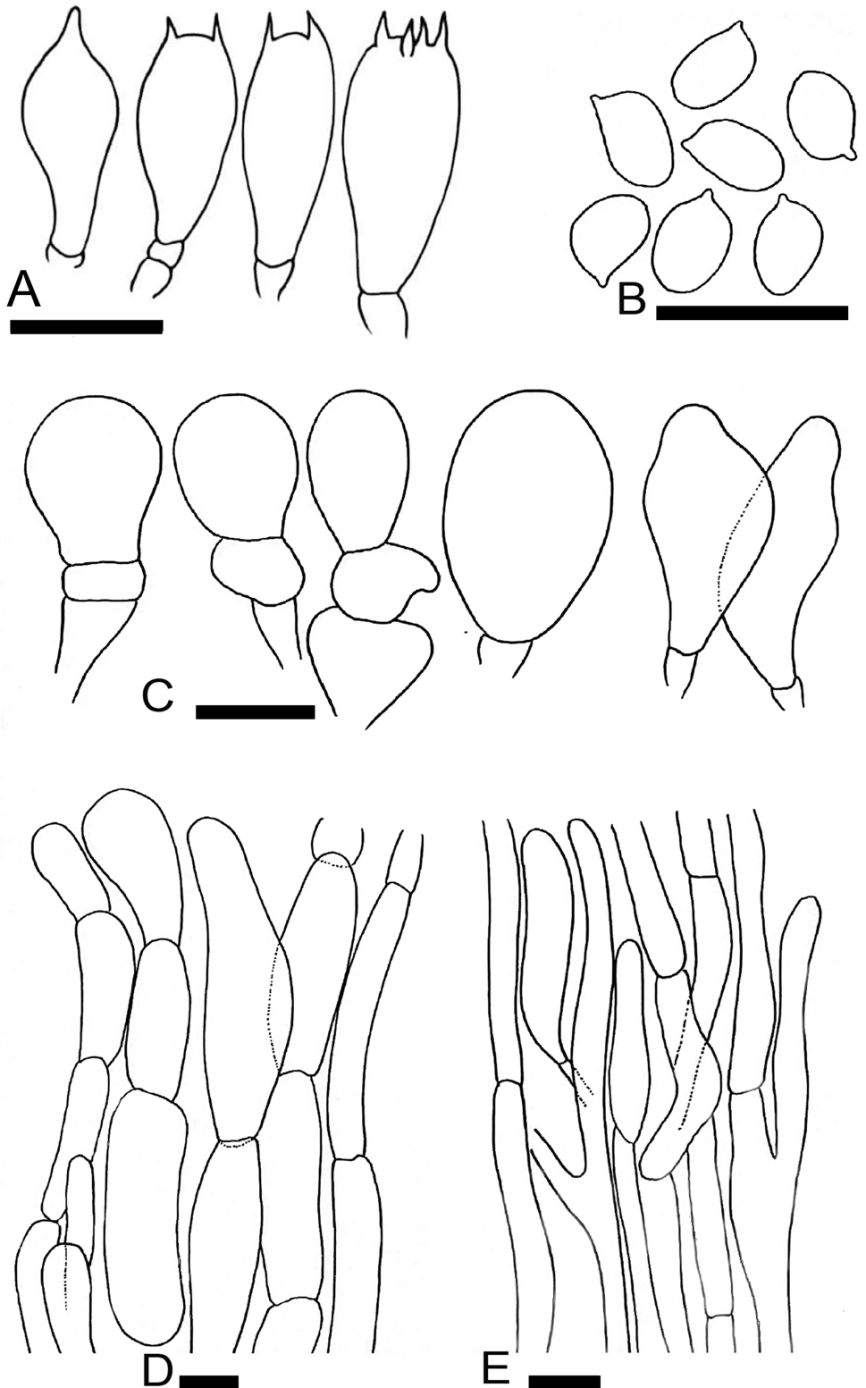
Micromorphological characters of *A.* *bambusetorum.* **(A–E)**  CM242 (**Holotype**); **(A)** Basidia, **(B)** Basidiospores, **(C)** Cheilocystidia, (**D)** Pileipellis hyphae, **(E)** Stipitipellis hyphae. Bars = 10 µm. Drawings by Dr. Hira Bashir.

***Agaricus*** ***bambusetorum*** H. Bashir & Niazi *sp.* *nov.* Figs. 4A–D, 5A–E

MycoBank: MB828314

**Main** **characters:** Morphologically, this taxon is characterized by small basidiomata having a white shiny pileus without scales, a simple narrow fragile annulus, a stipe discoloring brown upon rubbing and a faint yellow discoloration appearing slowly after cutting, a faint mushroom-like odor, predominantly bisporic basidia, and a subtropical distribution.

**Typification:** Pakistan, Punjab, Kasur District, Changa Manga forest, at 218 m a.s.l., on thick layer of dry leaf litter under Bamboo trees, 3 Sep 2016, A.R. Niazi & H. Bashir CM242 (**holotype** LAH35351). GenBank: ITS = MH923515; LSU = MK100288; TEF1 = MK169410.

**Etymology:** The specific epithet *bambusetorum* means *Agaricus* of the bamboo forests. From the Latin name of the bamboo (*Bambusa*) and the suffix “-*etorum*” (genitive of “-*etum*”) meaning “of the place dominated by”.

**Species-specific** **ITS** **markers:** tctggTtgggt@19, gaagt[T]ggtca@120, tcttt[–]gcctatcagGgtcta@[201-202]-211, caactTtcctg@460 and ccagtCtCatggg@635-638. The two latter (C) are within the insertion of 7 bp characterizing *A.* sect. *Hondenses*.

**Original** **description:** *Pileus* 2.5–3.5 cm in diam., hemispheric to convex, surface white (5.8 PB 9/2.9), smooth (scales not observed on any specimen), shiny (more pronounced in NWL322). *Margin* entire in young sporocarps, splitting radially at maturity, appendiculate, not exceeding the lamellae. *Lamellae* 3–5 mm in diam., pinkish-brown (2.1R 5.6/2) to dark brown (7.5YR 3/1.9), free, crowded, intercalated with lamellulae and with entire edges. *Stipe* 3–9.5 × 0.5–1 cm, cylindrical with bulbous base (1.5–2.5 cm wide), usually curved in the lower half, fistulose, provided with annulus in its upper part, above and below the annulus smooth and white (4.6 PB 8.7/3) with brown (6.5R 6.7/0.9) tinge with age and becoming brown immediately when rubbed/touched. *Annulus* superous, simple, narrow, membranous, fragile, white (4.9 PB 9/3.1) and smooth in both sides. *Context* when cut white, with a faint yellow discoloration appearing slowly. *Odor* faint, typical mushroom-like, pleasant.

*Basidiospores* (4.0–) 5.0–6.9 (–7.7) × (2.5–) 3.0–5.2 (–5.7) µm, [avX = 6.0 ± 0.47 × 4.1 ± 0.33 µm, Q_m_ = 1.49, n = 2 × 30], ellipsoid, light to dark brown in KOH, smooth, with a prominent apiculus, without apical pore and with granular content. *Basidia* 12–17 × 6–8 µm, broadly clavate or slightly truncate at the apex, hyaline in KOH, frequently bisporic rarely monosporic or tetrasporic. *Cheilocystidia* fusiform, broadly clavate, pyriform or ovoid, simple or septate at the base, terminal elements 12–25 × 7–19.5 µm, ante-terminal elements shortly cylindrical or globose. *Pleurocystidia* absent. *Underside* *of* *the* *annulus* not observed. *Pileipellis* hyphae 4–25 µm in diam., frequently septate, the wider the most constricted at the septa and with elements with rounded ends, branched. *Stipitipellis* hyphae 5–9 µm in diam., cylindrical, parallel, hyaline in KOH, smooth, branched.

**Macrochemical** **reactions:** KOH reaction positive, yellow. Schaeffer’s reaction—negative.

**Habit,** **habitat** **and** **distribution:** Growing solitary and/or in groups on grassy places under or near bamboo trees. Until now, it is known only from subtropical climate area in Pakistan.

**Additional** **specimens** **examined:** Pakistan, Punjab, Narowal, at 375 m a.s.l., solitary on rich loamy soil near bamboo trees, 14 Aug 2016, Humaira Bashir, NWL314 (LAH35352). GenBank: ITS = MH923516.

**Notes:** Morphologically, in *A*. sect. *Hondenses,* only *A.* *hondensis* sometimes has a white pileus surface but this species is very different from *A.* *bambusetorum* by having larger basidomata (pileus 8–15 cm), a broad annulus, a yellow discoloration of the context, a strong odor of phenol and smaller spores (5.1 × 3.4 µm on average). Many species in *A*. sect. *Xanthodermatei* also have a white pileus surface but their annulus is always membranous, thick and complex with a squamose lower surface or a double or triple edged margin. One among these, *A.* *pseudopratensis* (Bohus) Wasser, can sometimes have an annulus with simple margin but it differs by having a more robust habit, a context first discoloring pale yellow becoming blood red with time, an odor of phenol and predominantly tetrasporic basidia. *Agaricus* *parviniveus* (described in this study) also has a white pileus surface and predominantly bisporic basidia but it differs by having a squat habit, a triple edged annulus margin and shorter spores. Phylogenetically, the most closely related species *A.* *biannulatus* and *A.* *phaeolepidotus* (F.H. Møller) F.H. Møller have a squamulose pileus surface covered by ochraceous or brownish scales, thick complex annulus, different patterns of context discoloration and tetrasporic basidia. *Agaricus* *bambusetorum* is the only known non-temperate species in *A.* sect. *Hondenses*.

***Agaricus*** **sect.** ***Xanthodermatei*** Singer, Sydowia 2: 36. 1948

MycoBank: MB526821

Type: ***Agaricus*** ***xanthodermus*** Genev., Bull. Soc. Bot. France 23: 32. 1876

**Figure 6:**
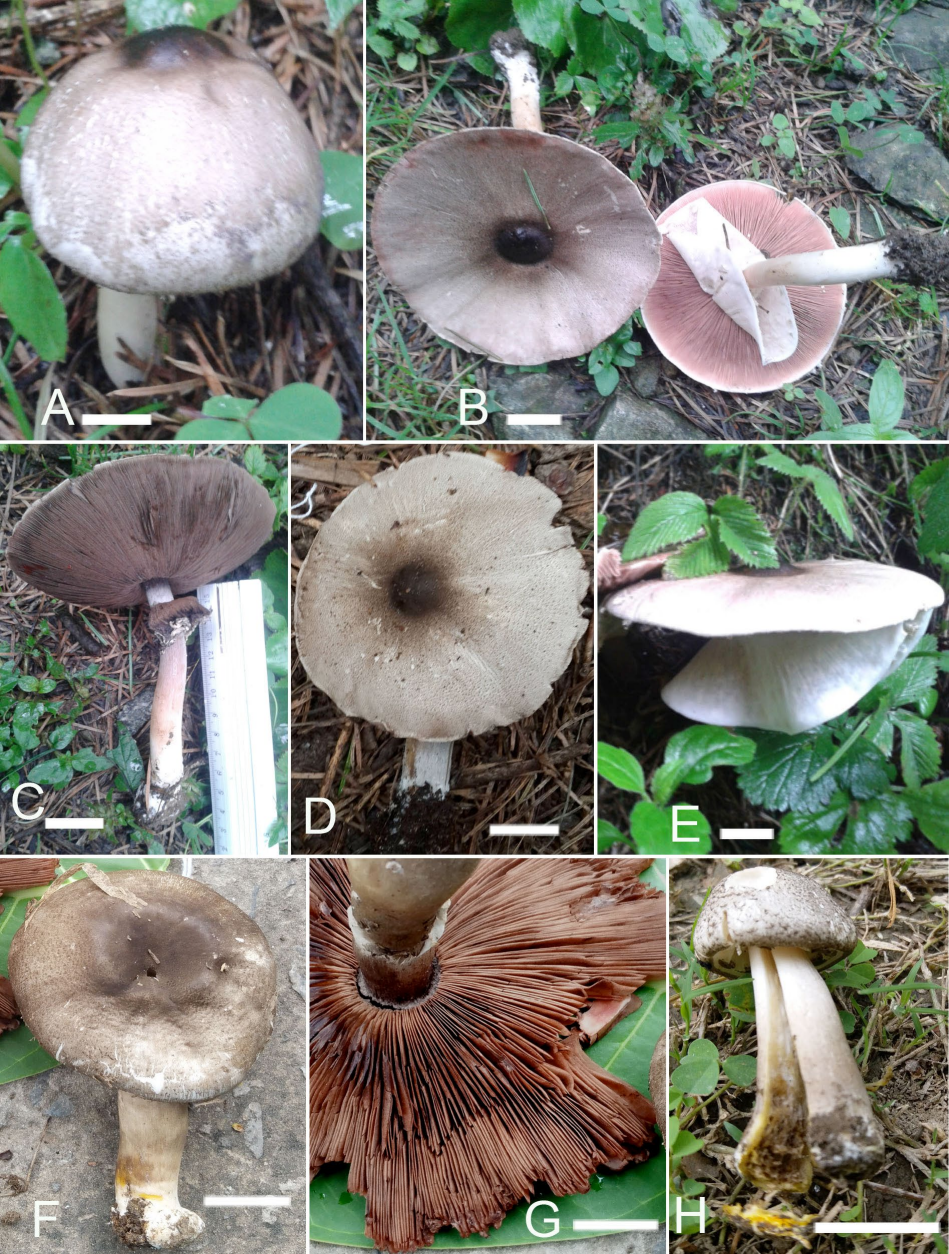
Macromorphological characters of *A.* *atroumbonatus* **(A–E)** and *A.* *fumidicolor* **(F–H)***.* **(A–H)** basidiomata in the field. **(A–C)**  MM26; **(D,E)**  MM1637 (**Holotype**); **(F–H)**  CM17 (**Holotype**). Bars = 1 cm. Photographed by Dr. Hira Bashir.

**Figure 7:**
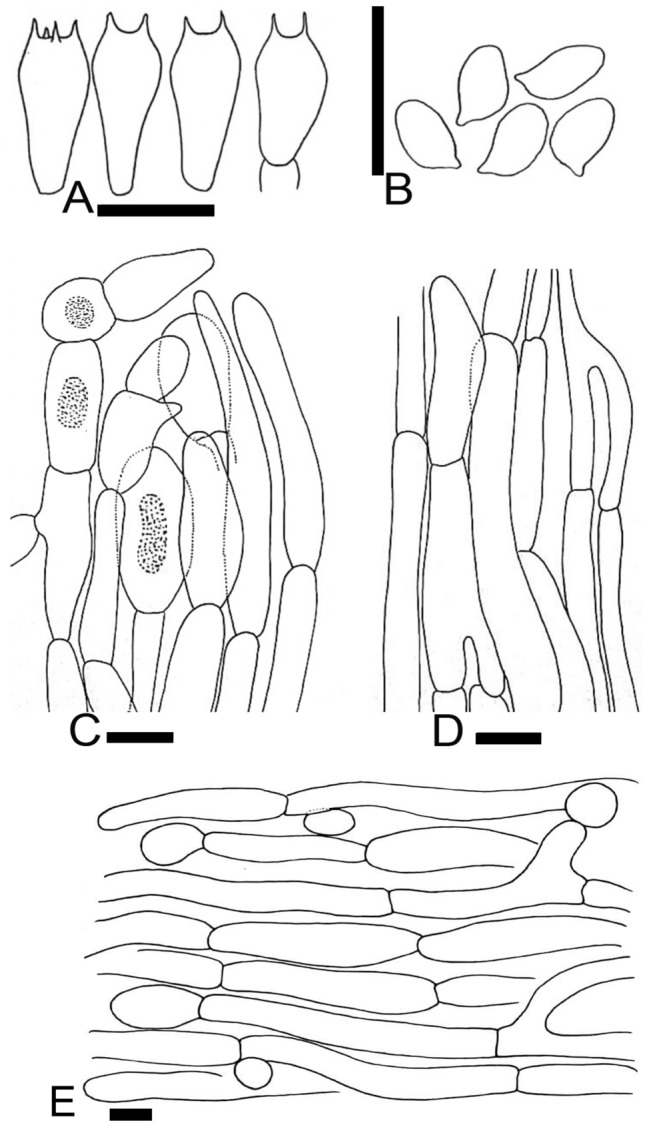
Micromorphological characters of *A.* *atroumbonatus.* **(A–E)**  MM1637 (**Holotype**). **(A)** Basidia, **(B)** Basidiospores, **(C)** Pileipellis hyphae, **(D)** Stipitipellis hyphae, **(E)** Hyphae of the underside of the annulus. Bars = 10 µm. Drawings by Dr. Hira Bashir.

***Agaricus*** ***atroumbonatus*** H. Bashir, J. Khan, Khalid, L.A. Parra & Callac, *sp.* *nov.* Figs. 6A–E, 7 A–E

MycoBank: MB828264

**Main** **characters:** This taxon is characterized by its pileus with blackish brown umbonate disc, thick annulus, whitish or brown stipe turning dirty grey after rain, faint phenolic odor, basidiospores measuring 4.8 × 2.8 µm on average, underside of the annulus with subglobose cells and basidia bisporic to tetrasporic.

**Typification:** Pakistan, Khyber Pakhtunkhwa, Swat District, Miandam valley, at 2200 m a.s.l., in decomposing needles under *Abies* *pindrow* Royle., 24 Aug 2014, Junaid Khan MM1637 (**holotype** LAH35353). GenBank: ITS = MH997905; LSU = MK100290; TEF1 = MK169412.

**Etymology:** The specific epithet is a compound Latin word where “*atro*” means dark colored and “*umbonatus*” refers to the umbonate shape of the pileus.

**Species-specific** **ITS** **markers—**gttgtGaagga@478 and tgcatCagcag@545.

**Original** **description:** *Pileus* 8–12 cm in diam., first hemispherical, then convex with incurved margin, later plane with straight margin and finally plano-concave, umbonate, dark brown to black and the umbo entirely covered by dark brown to blackish (2.9YR 1/0.8) squamules, dotted squamules on a white (5.9Y 8/0.7) background outside the umbo. *Surface* dry and dull. *Margin* entire in young sporocarps, eroded, slightly and wavy at maturity, vaguely exceeding lamellae. *Lamellae* pink (8.3RP 7/3.8) when young, becoming dark brown (2.3YR 4.3/2.2) at maturity, free, crowded, intercalated with lamellulae and entire edge. *Stipe* 10–12 × 1.3–1.5 cm, clavate to slightly bulbous at base, stuffed, provided with an annulus in its upper part near the lamellae, white to brown (9.9YR 4.6/3) and smooth to finely fibrillose above and below the annulus (in young sporocarp of MM26), dirty grey (0.2 PB 5.5/0.7) after rain from the base, immediately discoloring brown when rubbed. *Annulus* superous, double edged, thick, very broad, flaring, white (0.2B 9.6/0.7) in both sides, upper side radially striate, underside floccose. *Context* white when cut, becoming brown at the stipe. *Odor* of phenol, faint.

*Basidiospores* (4.0–) 4.5–5 (–5.5) × (2.5–) 2.7–2.9 (–3.2) µm, [avX = 4.8 ± 0.58 × 2.8 ± 0.21 µm, Q_m_ = 1.72, n = 2 × 30], ellipsoid to ellipsoid-elongate, light to dark brown in KOH, smooth with a prominent apiculus, without apical pore and with granular content. *Basidia* 10–17 × 5.5–8 µm, broadly clavate or slightly truncate at the apex, hyaline in KOH, bisporic to tetrasporic. *Cheilocystidia* absent. *Pleurocystidia* absent. *Underside* *of* *the* *annulus* consisting of two types of hyphae some cylindrical, not or slightly constricted at the septa, composed of elongated elements, 3–13 µm in diam., terminal elements with rounded ends; other consisting of globose to subglobose, ovoid or slightly clavate elements, narrow at septa, up to 17 µm wide. *Pileipellis* consisting of cylindrical hyphae 3–13.5 µm in diam., frequently septate and branched, very few subglobose cells also observed, the wider the most constricted at the septa, in KOH some hyaline and some with an internal brown vacuolar or diffuse pigment, terminal elements with rounded ends. *Stipitipellis* hyphae 3.5–10.5 µm in diam., cylindrical hyphae mixed with broader hyphae, parallel, hyaline in KOH, branched.

**Macrochemical** **reactions:** KOH reaction positive, yellow. Schaeffer’s reaction—negative.

**Habit,** **habitat** **and** **distribution:** Solitary or in pairs on grassy places under *Abies* *pindrow* Royle. It is known only from moist temperate areas of Pakistan so far.

**Additional** **specimen** **examined:** Pakistan, Khyber Pakhtunkhwa, Swat District, Miandam valley, at 2200 m a.s.l., in decomposing needles under *Abies* *pindrow*, 1 Aug 2014, Sadiq Ullah, MM26 (LAH35354). GenBank: ITS = MH997904.

**Notes:** *Agaricus* *atroumbonatus* shares most of the morphological characters with other members of clade Xan III. Therefore, molecular methods are crucial to distinguish it from other species of this clade. In both ITS and multi-gene phylogenetic analyses, *A.* *atroumbonatus* is sister to *A.* *daliensis* H.Y. Su & R.L. Zhao with strong statistical support in multi-gene tree (BS = 99, PP = 1, Fig. 2). Morphologically *A.* *daliensis* only differs from *A.* *atroumbonatus* by exhibiting yellow discoloration at the stipe base when cut. In addition, the ITS sequences of these two species differ at eight positions. To a lesser extent, *Agaricus* *atrodiscus* L.J. Chen, Callac, R.L. Zhao & K.D. Hyde is also phylogenetically related to *A.* *atroumbonatus*, but this species is tropical and differs by having an strong odor of phenol, bigger spores on average (5.2 × 3.3 µm) and abundant broadly clavate to sphaeropedunculate cheilocystidia.

**Figure 8:**
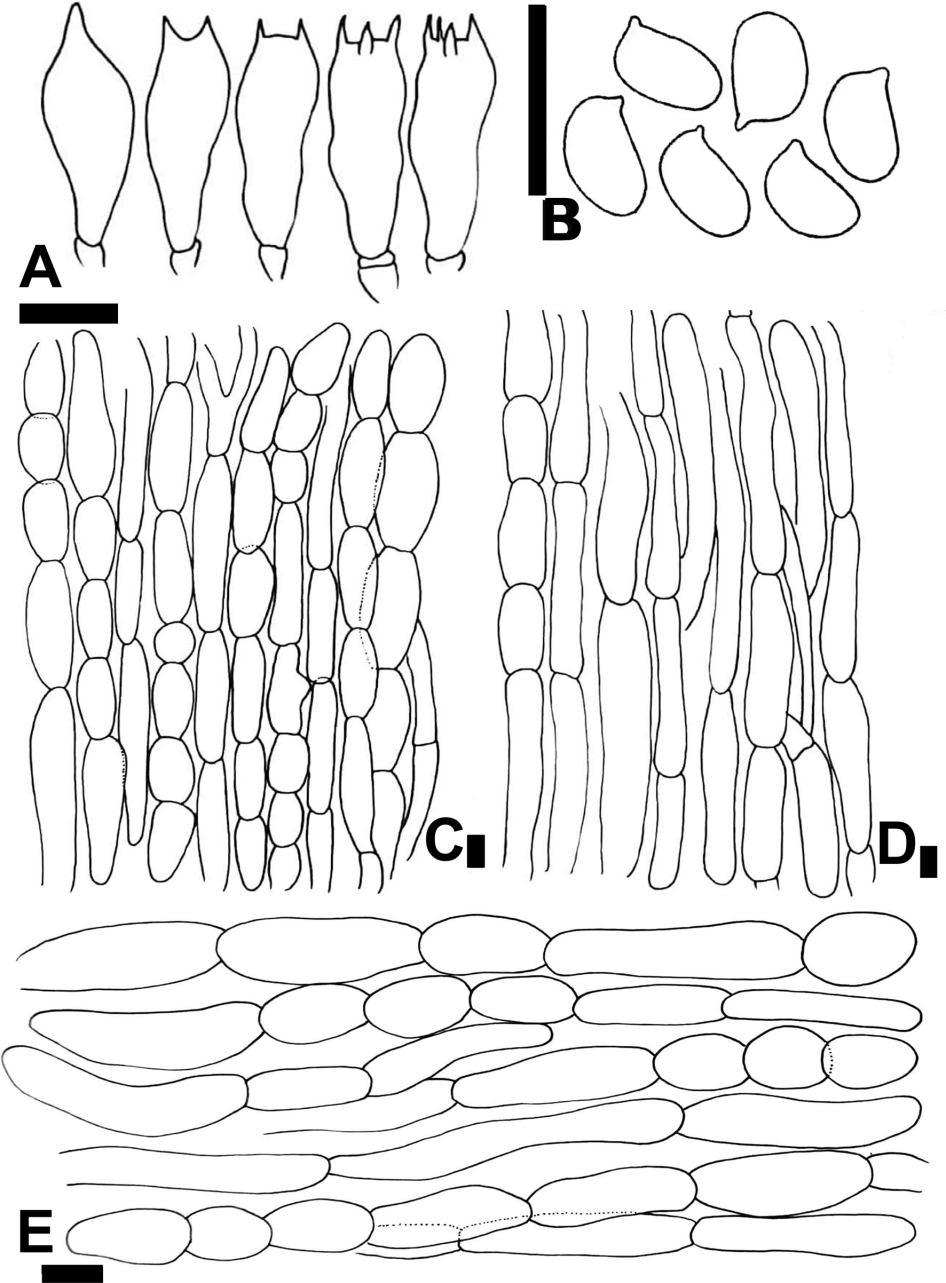
Micromorphological characters of *A.* *fumidicolor.* **(A–E)**  CM17 (**Holotype**). **(A)** Basidia, **(B)** Basidiospores, **(C)** Pileipellis hyphae, **(D)** Stipitipellis hyphae, **(E)** Hyphae of the underside of the annulus. Bars = 10 µm. Drawings by Dr. Hira Bashir.

***Agaricus*** ***fumidicolor*** H. Bashir, Niazi, Khalid & L.A. Parra, *sp.* *nov.* Figs. 6F–H, 8A–E

MycoBank: MB 818952

**Main** **characters:** This species is characterized by a dark, blackish grey pileus, a light greyish brown color on the stipe, basidiospores 5.7 × 3.9 µm on average, basidia mostly bisporic and cheilocystidia absent.

**Typification:** Pakistan, Punjab, Kasur, Changa Manga forest, at 214 m. a.s.l., gregarious on forest ground near bamboo trees, 02 Aug 2015, H. Bashir & A.R. Niazi, CM17 (**holotype** LAH35196). GenBank: ITS = KY751305.

**Etymology:** The specific epithet ‘*fumidicolor’* refers to the smoky (*fumidus* in Latin) color of the pileus.

**Species-specific** **ITS** **markers:** atggaAgaagt@114, tatcaActcccgGtggat@135-142, cagtgGgaaagcag[C]tgGtgtccgcta[T]cttgg@164-173-176-186, tgctcCcttgGgtCcagct@611-616-619 and aatcgCcttcattgCcaatt@633-642.

**Original** **description:** *Pileus* 8–11.5 cm in diam., hemispherical then convex with depressed centre at maturity, entire and blackish grey (5YR2/2) at disc, outside the disc densely covered with concolorous punctiform squamules progressively scattered towards the margin, giving a smoky appearance to the pileus, on a white background. *Surface* dry and dull. *Margin* entire, slightly splitting at maturity. *Lamellae* 3–6 mm in diam., at first light brown, then dark brown (7.5YR1/2), free, crowded, with intercalated lamellulae and with entire edges. *Stipe* 8.4 × 1.4–1.7 cm wide at maturity, cylindrical, slightly bulbous at base when mature, sometimes curved, solid, provided with an annulus in its upper part, above the annulus smooth and white, below smooth, light greyish brown in its lower half and white in the extreme base, rhizomorphs present. *Annulus* superous, broad, fragile, upper side smooth, underside with perimarginal linear dark grey squames (apparent in young basidiomata), white. *Context* white, discoloring bright yellow immediately when cut, more pronounced in young basidiomata becoming light pinkish-brown with time. *Odor* strong of phenol.

*Basidiospores* (4.6–) 5.0–6.2 (–6.8) × (3.2–) 3.6–4.0 (–4.9) µm, [avX = 5.7 ± 0.55 × 3.9 ± 0.36 µm, Q_m_ = 1.45, n = 2 × 30], ellipsoid, smooth with a prominent apiculus, thick-walled, dark brown. *Basidia* 13–25 × 5.0–10 µm, clavate, mostly bisporic, some monosporic and trisporic, very rarely tetrasporic, hyaline in KOH, smooth. *Cheilocystidia* absent. *Pleurocystidia* absent. *Underside* *of* *the* *annulus* consisting of hyphae with short and elongated mixed elements 4–13 µm in diam., constricted at septa, hyaline in KOH, terminal elements with more or less rounded ends. *Pileipellis* a cutis made up of cylindrical hyphae 3–16 µm in diam., very frequently septate, with globose to doliiform elements distinctly constricted at the septa, with terminal elements with rounded tips, some hyaline and others with internal diffuse or vacuolar brown pigment in KOH. *Stipitipellis* constituted by hyphae 1.7–15.4 µm in diam., hyaline in KOH, thin-walled, frequently septate, branched.

**Macrochemical** **reactions:** KOH reaction positive, bright yellow. Schaeffer’s reaction negative.

**Habit,** **habitat** **and** **distribution:** Growing in open clusters on nutrient rich loamy soil near bamboo trees. It is only known from the plains of southeast Pakistan so far.

**Additional** **specimens** **examined:** Pakistan, Punjab, Kasur, Changa Manga forest, at 214 m. a.s.l., gregarious on rich loamy soil near bamboo trees, 3 Sep 2016, H. Bashir & A.R. Niazi, CM100, LAH35217. GenBank: ITS = KY741306.

**Notes:** As for the species described above (*A.* *atroumbonatus*), an unequivocal morphological differentiation with all taxa of clade Xan III is not possible. In the ITS tree (Fig. 2), *A.* *fumidicolor* clusters with three other tropical or subtropical species in a well-supported clade (BS = 99%, PP = 1). In this clade *A.* *fumidicolor* is sister to *A.* *punjabensis* and is also related but to a lesser extent to *A.* *endoxanthus* and but these two species differ from *A.* *fumidicolor* by having smaller spores on average (4.5 × 3.2 µm and 5.2 × 3.3 µm, respectively) and clavate to sphaeropedunculate cheilocystidia.

***Agaricus*** ***gregariomyces*** J.L. Zhou & R.L. Zhao, Phytotaxa 257 (2): 111. 2016 Figs. 9A, 10A–F.

**Figure 9:**
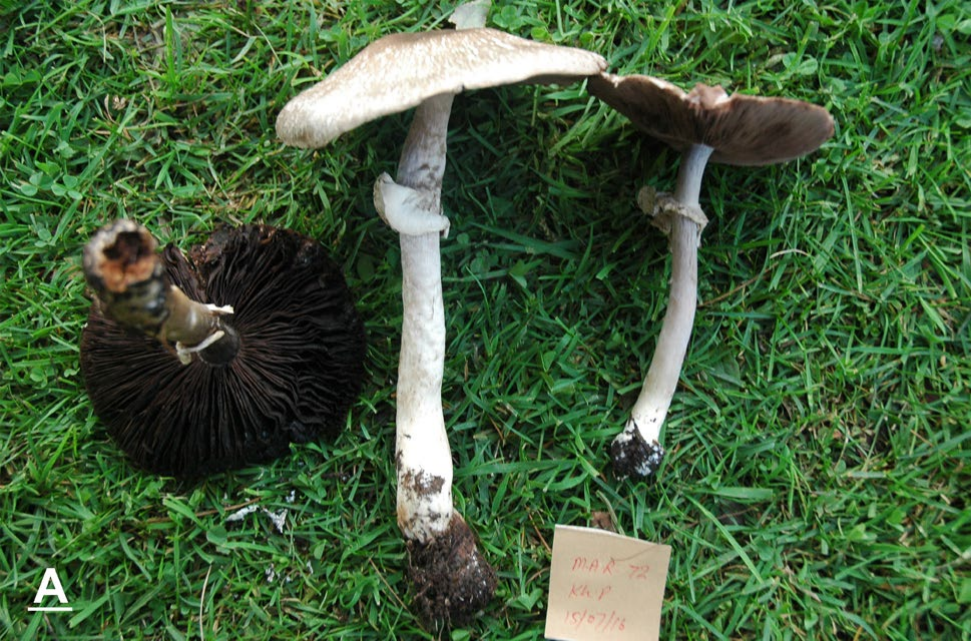
Macromorphological characters of *A.* *gregariomyces.* A basidiomata in the field. **(A)**  KH72. Bars = 1 cm. Photographed by Dr. Hira Bashir.

**Figure 10:**
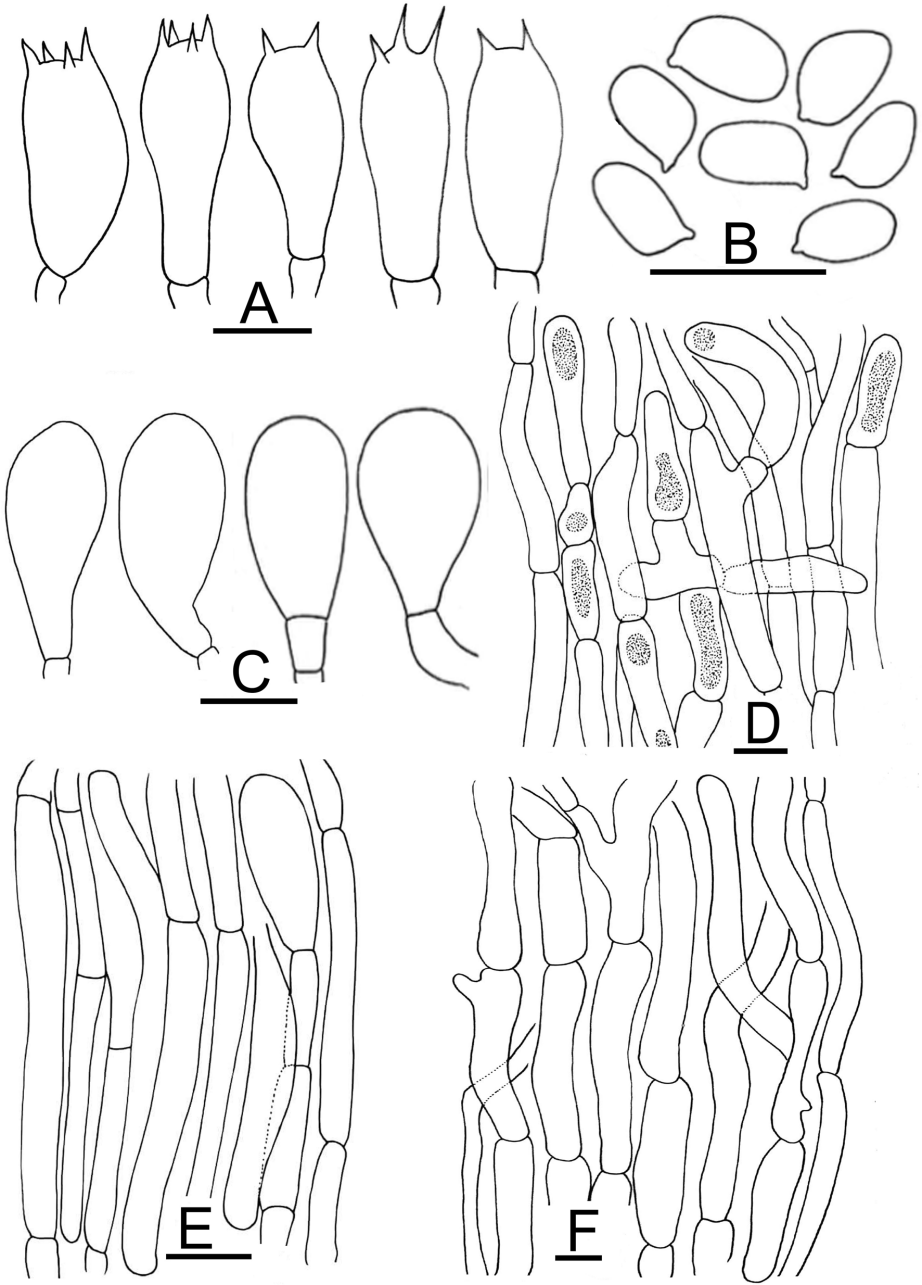
Micromorphological characters of *A.* *gregariomyces.* **(A–F)** = KH72. A Basidia, **(B)** Basidiospores, **(C)** Cheilocystidia, **(D)** Pileipellis hyphae, **(E)** Stipitipellis hyphae, **(F)** Hyphae of the underside of the annulus. Bars = 10 µm. Drawings by Dr. Hira Bashir.

MycoBank: MB812314

**Description:** *Pileus* 6–8.5 cm in diam., conical in youngsporocarps, then truncate conical, completely covered with greyish brown scales radially arranged. *Surface* dry and dull. *Margin* slightly recurved not exceeding the lamellae. *Lamellae* free, crowded, intercalated with lamellulae, dark brown. *Stipe* 6–10.5 × 0.5–1.5 cm, clavate to cylindrical slightly bulbous at base, stuffed, provided with rhizomorphs at the base and an annulus in its upper third, smooth to slightly fibrillose and white above and below the annulus, discoloring light brown when touched towards the base. *Annulus* superous, double, white, flaring, broad, upper side smooth, underside floccose, with a double-edged margin. *Context* discoloring faint yellow when bruised. *Odor* of phenol, mild.

*Basidiospores* (5.0) 5.6–6 (–6.5) × (2.8–) 3.4–3.9 (–4.3) µm [x = 5.77 ± 0.5 × 3.5 ± 0.7 µm, Q_m_ = 1.67, n = 1 × 30], ellipsoid to ellipsoid-elongate mostly, dark brown at maturity, smooth with a prominent apiculus and without apical pore. *Basidia* 9.0–23 × 8–13.5 µm, clavate to broadly clavate or slightly truncate at the apex, hyaline in KOH, frequently bisporic to tetrasporic. *Cheilocystidia* abundant, usually simple, clavate to broadly clavate, some sphaeropedunculate, with terminal elements 11.5–23 × 4.0–15 µm clavate to pyriform and ante-terminal elements, when present, cylindrical. *Pleurocystidia* absent. *Underside* *of* *the* *annulus* consisting of cylindrical hyphae, 2.5–8 µm, slightly constricted at septa, the broader the most frequently septate, and terminal elements with rounded ends. *Pileipellis* consisting of hyphae 3–8 µm in diam., frequently septate and branched, slightly constricted at septa, mostly with internal brown pigmentation and some hyaline in KOH, terminal elements with rounded ends. *Stipitipellis* hyphae 3.5–7.5 µm in diam., cylindrical with some broader elements, arranged parallelly, rarely branched, hyaline in KOH.

**Macrochemical** **reactions:** KOH reaction positive, light yellow. Schaeffer’s reaction negative on dry basidiomata.

**Habit,** **habitat** **and** **distribution:** Gregarious on grassy ground with leaf litter near pine trees. It is known from China and Pakistan.

**Specimen** **examined:** Pakistan. Khyber Pakhtunkhwa, Khanspur, at 2250 m a.s.l., 15 Jul 2016, Muhammad Ali & A.R. Niazi KH72 (LAH35795). GenBank: ITS = MK101032.

**Notes:** *Agaricus* *gregariomyces*, originally described from China from a single collection (ZRL2012624), is reported for the first time from Pakistan (KH72). Macroscopic characters of both collections match except for the odor described as pleasant in the Chinese collection and mild phenol-like in the Pakistani collection. Microscopically, spore size is similar in both collections, however only tetrasporic basidia were observed in ZRL2012624 while frequent bisporic or tetrasporic basidia but rare trisporic basidia were observed in KH72. In addition, cheilocystidia were not observed in ZRL2012624 while they are abundant, broadly clavate to sphaeropedunculate shaped in KH72. *Agaricus* *gregariomyces* shares most of the morphological characters with other members of *A*. sect. *Xanthodermatei*. Therefore, molecular methods are crucial to identify this species. In this clade, the most related species are *A.* *buckmacadooi* Kerrigan and *A.* *kriegeri* Kerrigan, which are known only in North America and not specially at high altitude contrarily to *A.* *gregariomyces* which was collected in Tibet in China and at Khanspur (average altitude 2250 m a.s.l.) in Pakistan. In addition, *A.* *buckmacadooi* and *A.* *kriegeri* differ by having a smooth pileus surface and spores shorter and wider (Q = 1.44–1.45). Also, *A.* *buckmacadooi* has a narrow annulus.

**Figure 11:**
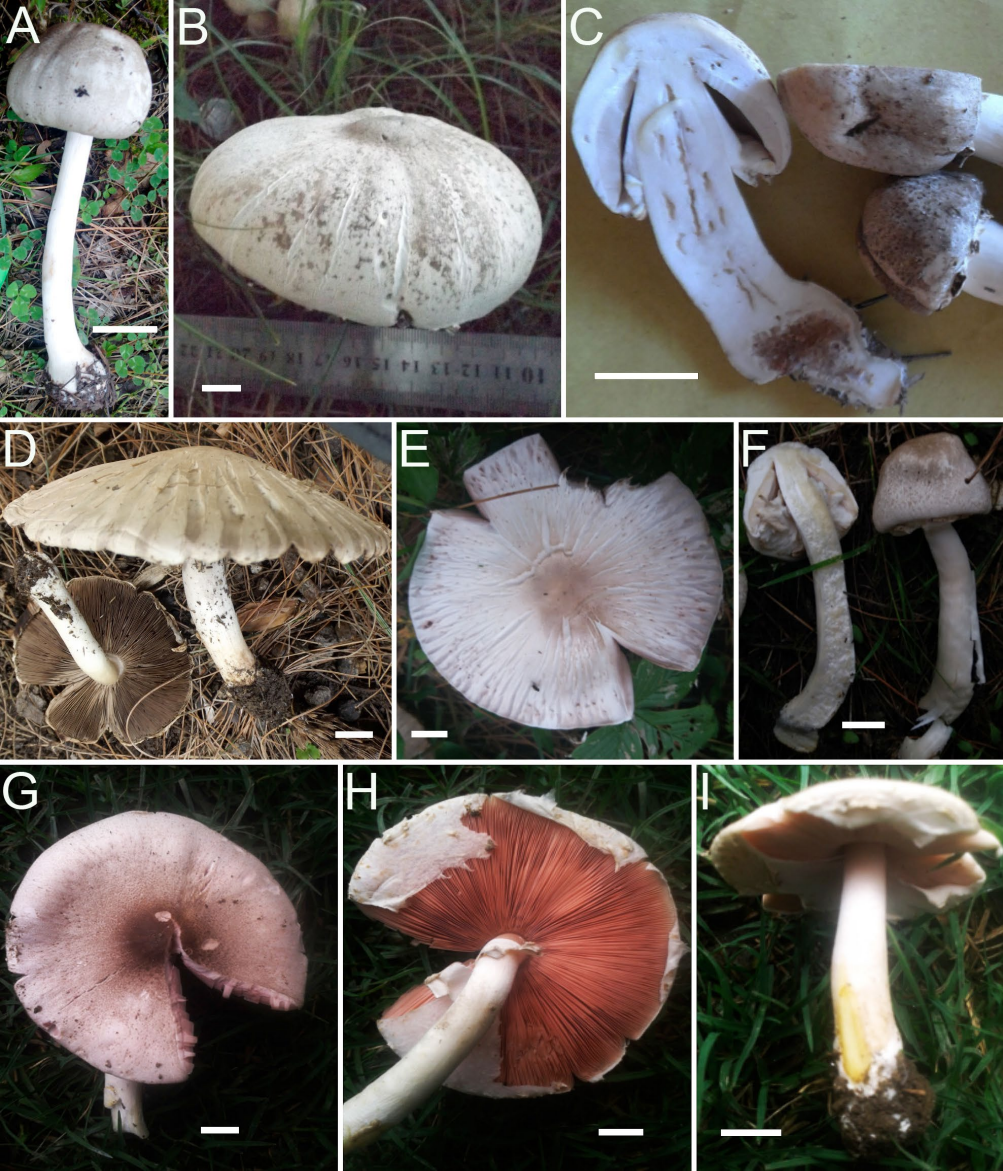
Macromorphological characters of *A.* *griseovariegatus* **(A–F)** and *A.* *macropeplus* **(G–I)***.* **(A–I)** Basidiomata in the field. **(A)**  BG37; **(B)**  SA194; **(C)**  SA850; **(D)**  SA232; **(E**,**F)**  KH167 (**Holotype**); **(G,H)**  MW1501 (**Holotype**); **(I)**  MW1544. Bars = 1 cm. Photographed by Dr. Hira Bashir.

**Figure 12:**
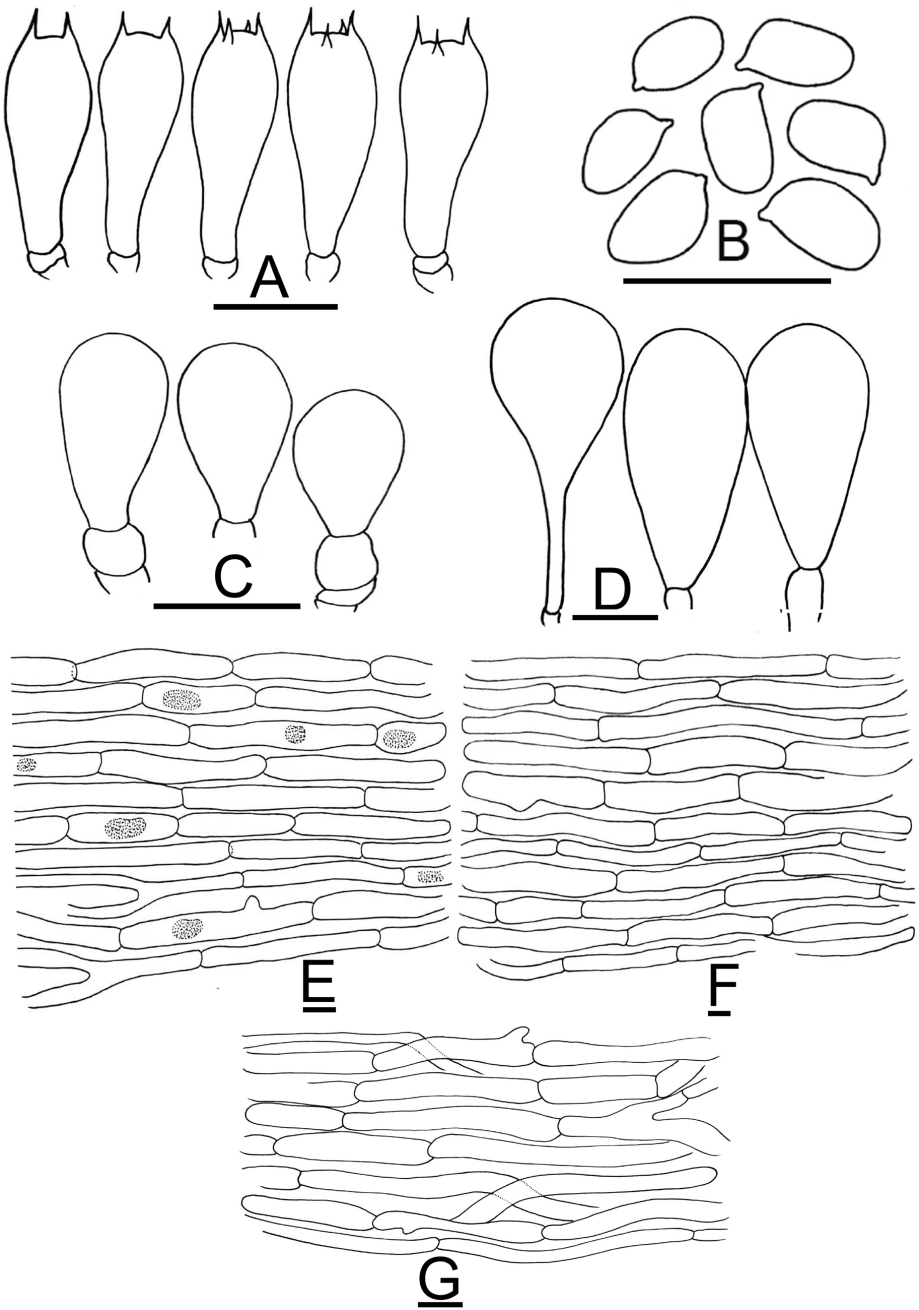
Micromorphological characters of *A.* *griseovariegatus.* **(A–G)**  KH167 (**Holotype**). **(A)** Basidia, **(B)** Basidiospores, **(C,D)** Cheilocystidia, **(E)** Pileipellis hyphae, **(F)** Stipitipellis hyphae, **(G)** Hyphae of the underside of the annulus. Bars = 10 µm. Drawings by Dr. Hira Bashir.

***Agaricus*** ***griseovariegatus*** H. Bashir, S. Ullah & Khalid, *sp.* *nov.* Figs. 11A–F, 12A–G.

MycoBank: MB 818983

**Main** **characters:** This taxon is characterized by its pileus with olive grey appressed squamules and broadly radially fissured at maturity, lower side of annulus floccose, context with a faint yellow discoloration, odor of phenol and bisporic to tetrasporic basidia.

**Typification:** Pakistan**,** Khyber Pakhtunkhwa, Khanspur, at 2250 m a.s.l., under *Pinus* *wallichiana* A.B. Jacks., gregarious, on decomposing needles, 26 Jul 2017, H. Bashir & Muhammad Ali, KH167 (**holotype** LAH35355). GenBank: ITS = MK101038; LSU = MK100284; TEF1 = MK169406.

**Etymology:** The specific epithet ‘*griseovariegatus’* refers to the variegated grey color of pileus.

Species-specific ITS markers—None.

**Original** **description:** *Pileus* 8–13.5 cm in diam., ovate to broadly parabolic when young, then convex to plano-convex, umbonate, covered with variegated olive-grey (5Y 3/2) appressed squamules in young stages turning dark olive grey (2.5Y 2/2) at maturity on a white background (10YR9/2), in the collections SA194, SA232, KH167 broadly radially fissured at maturity. *Surface* dry and dull. *Margin* entire in young sporocarps, then ruptured at maturity, slightly appendiculate, vaguely incurved, slightly exceeding the lamellae, yellowish-brown when rubbed. *Lamellae* white to light pinkish (8.3RP 7/3.8) at young stage and finally brown (7.5YR7/2) at maturity, free, crowded, intercalated with lamellulae and with entire edges. *Stipe* 10–15 × 2.0–2.6 cm at the apex, 2.5–3.0 cm at the bulbous base, cylindrical, stuffed, provided with annulus in its upper half close to lamellae, above and below the annulus smooth, white when young discoloring rusty brown by rubbing, becoming slightly brownish at maturity with rhizomorphs at the base in most specimens. *Annulus* superous, double edged, broad, pendant, membranous, upper side smooth, underside floccose, white with light brown perimarginal linear squamules. *Context* white, discoloring very faint yellow when cut in the upper half of the stipe (only in KH167). *Odor* of phenol.

*Basidiospores* (4.2–) 5.0–5.4 (–6.1) × (3.0–) 3.2–3.8 (–4.0) µm, [avX = 5.2 ± 0.46 × 3.6 ± 0.28 µm, Q_m_ = 1.46, n = 4 × 30], ellipsoid, light to dark brown in KOH, smooth with a prominent apiculus. *Basidia* 14–22 × 5.0–7.0 µm, clavate, slightly truncate at apex, hyaline in KOH, frequently bisporic to tetrasporic. *Cheilocystidia* abundant, simple or septate at the base, with terminal elements 12.5–40.5 × 9.5–19 µm, broadly clavate to sphaeropedunculate and ante-terminal elements usually cylindrical, sometimes sub-globose, hyaline in KOH. *Pleurocystidia* absent. *Underside* *of* *the* *annulus* consisting of cylindrical hyphae, 3–8 µm in diam., branched, frequently septate and terminal elements with rounded ends. *Pileipellis* constituted by interwoven hyphae 4–10.5 µm in diam., septate, branched, not or slightly constricted at septa, with internal brown vacuolar pigmentation or hyaline in KOH, terminal elements with rounded ends. *Stipitipellis* constituted by hyphae 5.5–14 µm in diam., cylindrical, frequently septate, rarely branched, hyaline in KOH, the shorter and broader the more constricted at the septa, with terminal elements having rounded ends.

**Macrochemical** **reactions:** KOH reaction positive, bright yellow. Schaeffer’s reaction negative.

**Habit,** **habitat** **and** **distribution:** Solitary or gregarious in grassy places with mixed coniferous trees. It is only known from moist temperate areas of Pakistan so far.

**Additional** **specimens** **examined:** Pakistan, Khyber Pakhtunkhwa, Shangla district, Takht, on mountain top in grassy field, in sandy soil under *Pinus* *roxberghii* Sarg. and *Abies* *pindrow* Royle at 2500 m a.s.l., 27 Jul 2014, Sadiq Ullah SA194 (LAH35290). GenBank: ITS = MG669249; Khyber Pakhtunkhwa, Chakesar banda, in sandy soil under *Abies* *pindrow* Royle, at 2300 m a.s.l., 14 Aug 2015, Sadiq Ullah SA850 (LAH35291). GenBank: ITS = MG669250; Khyber Pakhtunkhwa, Shangla district, Takht, on mountain top in grassy field at 2500 m a.s.l., 2 Jul 2014, Sadiq Ullah SA232 (LAH35292). GenBank: ITS = MK101037. Azad Jammu & Kashmir, District Bagh, at 2400 m a.s.l., solitary in grassy place near coniferous trees, 28 Jul 2015, A.N. Khalid & R. Khurshid, BG37 (LAH35197). GenBank: ITS = KY741891; LSU = MK100285; TEF1 = MK169407.

**Notes:** Morphologically, *A.* *griseovariegatus* is characterized by its pileus with appressed squamules with olivaceous tones, and by its broadly radially fissured pileus margin at maturity. These two combined characters are not noticed neither in its sister species *A.* *xanthochromaticus* (Figs. 1 and 2) nor in other species of clade III. In clade II, the collection JBSD127423 from Dominican Republic similarly exhibits a pileus completely covered with dark greyish-brown squamules with olivaceous tones, but it is neither variegated nor radially fissured and lacks odor of phenol.

**Figure 13:**
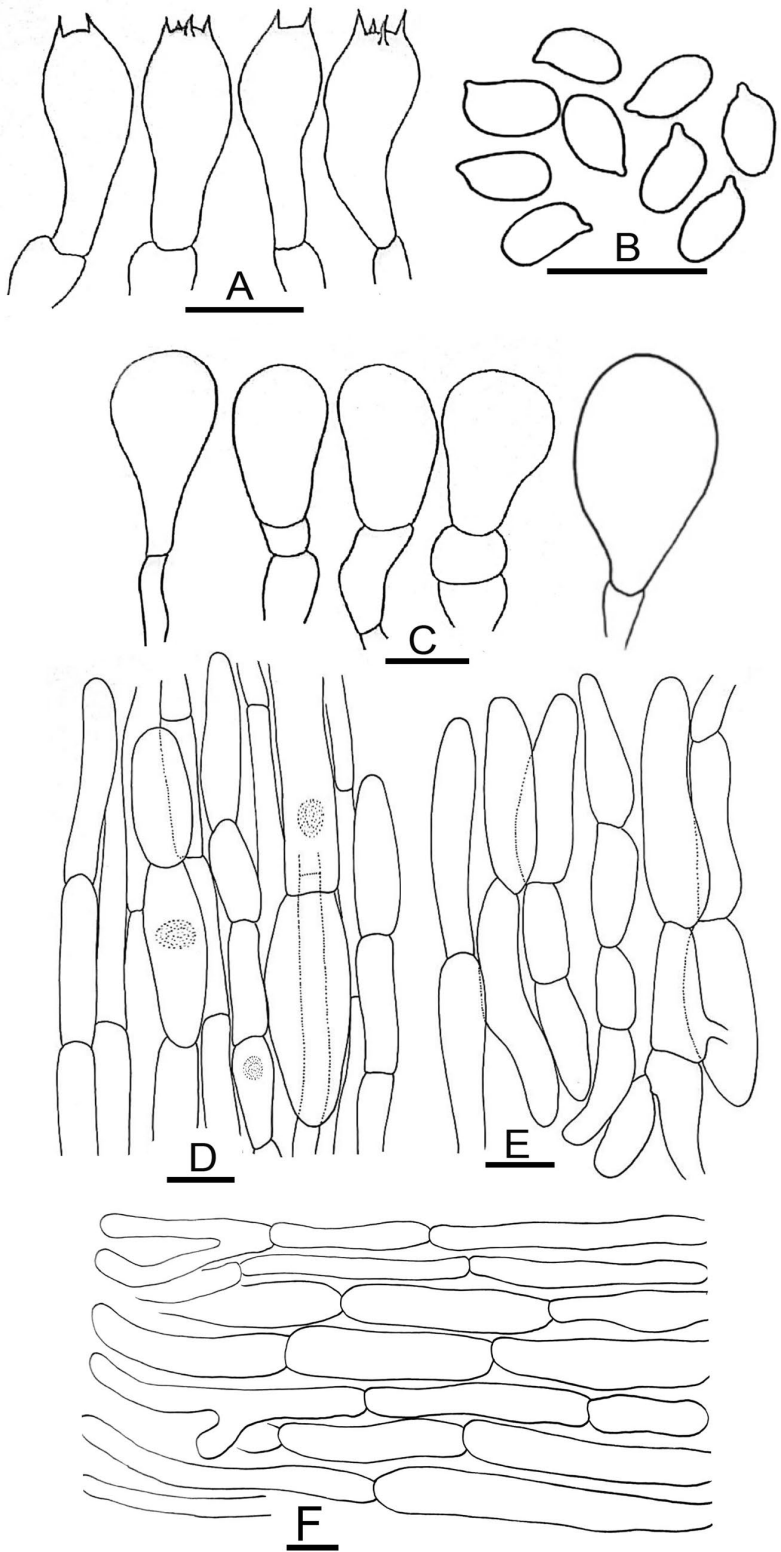
Micromorphological characters of *A.* *macropeplus.* **(A–F)**  MW1501 (**Holotype**). **(A)**  Basidia, **(B)**  Basidiospores, **(C)**  Cheilocystidia, **(D)**  Pileipellis hyphae, **(E)**  Stipitipellis hyphae, (**F) **  Hyphae of the underside of the annulus. Bars = 10 µm. Drawings by Dr. Hira Bashir.

***Agaricus*** ***macropeplus*** H. Bashir, J. Khan, Khalid & L.A. Parra, *sp.* *nov*. Figs. 11G–I, 13A–F

MycoBank: MB828265

**Main** **characters:** This species is characterized by a large pileus with brown dotted scattered squamules condensed at the umbonate disc, pileus frequently fissured in young and mature sporocarps, appendiculate margin with large remnants of veil, basidiospores 5.4 × 3.1 µm on average and basidia bisporic or tetrasporic.

**Typification**: Pakistan, Khyber Pakhtunkhwa, Swat District, Manglor, at 987 m a.s.l., in grass under deciduous trees, 14 Aug 2015, Junaid Khan, MW1501 (**holotype** LAH35356). GenBank: ITS = MK141002; LSU = MK100286; TEF1 = MK169408.

**Etymology:** The specific epithet “*macropeplus*” means broad annulus. From the Latinized Greek words *makros* (long, large, broad) and *peplos* (robe, tunic).

Species-specific ITS marker—None.

**Original** **description:** *Pileus* 5–9 cm in diam., convex to plano-convex, disc umbonate with brown coloration (7.2R 4/2). brown (2.2R 5.9/2.4) with dotted squamules covering the pileus completely, dense at disc, scattered towards margin, background white (7.5P 9/2.1). *Surface* dry and dull. *Margin* appendiculate, splitting in young and mature sporocarps, slightly exceeding lamellae. *Lamellae* 3–5 mm broad, light pink to brown (9.1R 5.9/6.2), free, crowded, intercalated with lamellulae with entire edges. *Stipe* 5–8 × 0.6–1 cm, cylindrical with slightly bulbous base, broad, stuffed, provided with an annulus in its upper half near lamellae, white (9.4R 8.7/0.9), light pinkish or brown (7.9R 6.9/3.8) from top, smooth above and below annulus, immediately discoloring light yellow when scratched. *Annulus* superous, double edged, white, flaring, broad, fragile, upper side radially finely striate, underside floccose. *Context* white initially becoming yellow immediately when cut. *Odor* of phenol, faint.

*Basidiospores* (4.6–) 5.3–5.5 (–6.2) × (2.5–) 2.9–3.4 (–3.6) µm, [avX = 5.4 ± 0.64 × 3.1 ± 0.47 µm, Q_m_ = 1.74, n = 2 × 30], ellipsoid, sometimes with an obscure supraapicular depression, hyaline in KOH, brown at maturity, smooth with a prominent apiculus and without apical pore. Along with the dark brown spores other ellipsoid-elongated bigger and paler spores can be frequently observed measuring (7.5–) 8–9.5 (–10.3) × (3.6–) 3.4–4.1 (–4.5) µm, [avX = 8.9 ± 1.36 × 4.0 ± 0.47 µm, Q_m_ = 2.20. *Basidia* 14–22 × 5.0–8.0 µm, hyaline in KOH, usually bisporic or tetrasporic much less frequently monosporic, clavate or slightly truncate at the apex. *Cheilocystidia* absent in MW1501, in MW1544 not very frequent, either clavate, 10–15 × 6–9.5 µm, septate at the base with ante-terminal elements frequently subglobose to more or less clavate, sometimes cylindrical or large-sized, simple, sphaeropedunculate, 22–32.5 × 11–13.5 µm. *Pleurocystidia* absent. *Underside* *of* *the* *annulus* consisting of hyphae 2.5–9 µm in diam., cylindrical or broader more constricted at the septa, branched, and terminal elements with rounded ends. *Pileipellis* constituted by cylindrical hyphae 4.0–16 µm in diam., frequently septate, branched, the wider the most constricted at the septa, with internal brown vacuolar pigmentation terminal elements with rounded tips. *Stipitipellis* hyphae 3–10 µm in diam., mostly cylindrical or broader more constricted at the septa with parallel arrangement, hyaline in KOH.

**Macrochemical** **reactions:** KOH reaction positive, yellow. Schaeffer’s reaction negative.

**Habit,** **habitat** **and** **distribution:** Growing solitary and in the group of two on grassy grounds near deciduous trees. The known distribution of this species is from deciduous forest of Swat, Pakistan until now.

**Additional** **specimen** **examined:** Pakistan, Khyber Pakhtunkhwa, Swat District, Manglor, at 987 m a.s.l., in grass near deciduous trees, 7 Sep 2015, Junaid Khan, MW1544 (LAH35357). GenBank: ITS = MK141003.

**Notes:** *Agaricus* *macropeplus* is morphologically very similar to many species of clade Xan III and therefore an unequivocal morphological differentiation with all taxa is not possible. Phylogenetically, *Agaricus* *macropeplus* clusters in the same clade as *A.* *griseovariegatus*, *A.* *sinoplacomyces* and *A.* *xanthochromaticus* (Figs. 1 and 2) from which it differs in the ITS sequence at 3, 5 and 6 nucleotide sites, respectively (Table 2). *Agaricus* *griseovariegatus* differs by having a pileus surface covered with brown squamules with olicaceous tones progressively scattered towards the margin but it is very difficult to distinguish *A.* *macropeplus* from *A.* *sinoplacomyces* and *A.* *xanthochromaticus*. *Agaricus* *sinoplacomyces* was described with a pleasant odor (but this feature needs confirmation) and *A.* *xanthochromaticus* has an intense odor of phenol and slightly smaller spores on average (4.9 × 3.3 µm).

**Figure 14:**
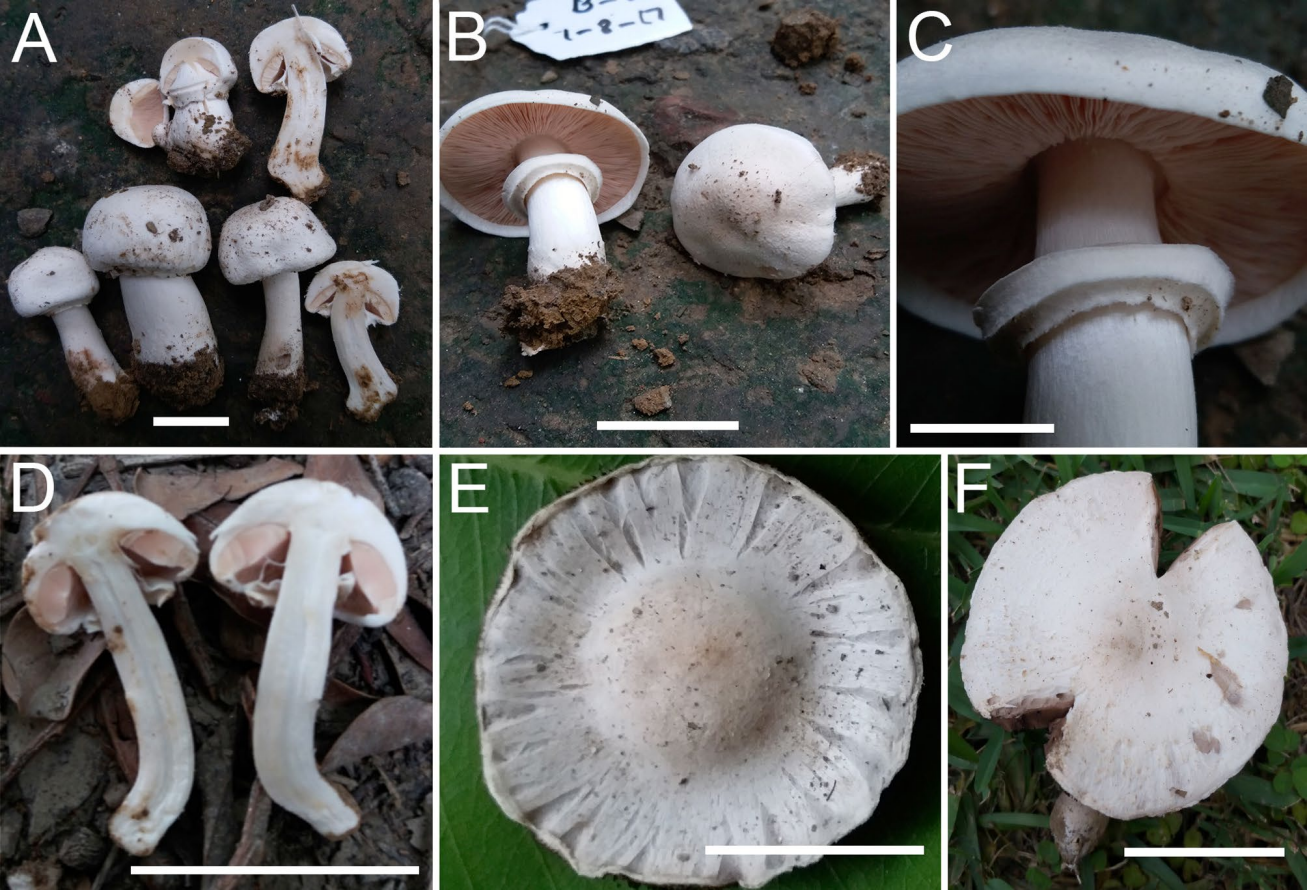
Macromorphological characters of *A.* *parviniveus.* **(A–F)** basidiomata in the field. **(A–D) **  B40 (**Holotype**); **(E)** PU318; **(F)**  PU248. Bars*:* **(A–F)** = 2 cm. Photographed by Dr. Hira Bashir.

**Figure 15:**
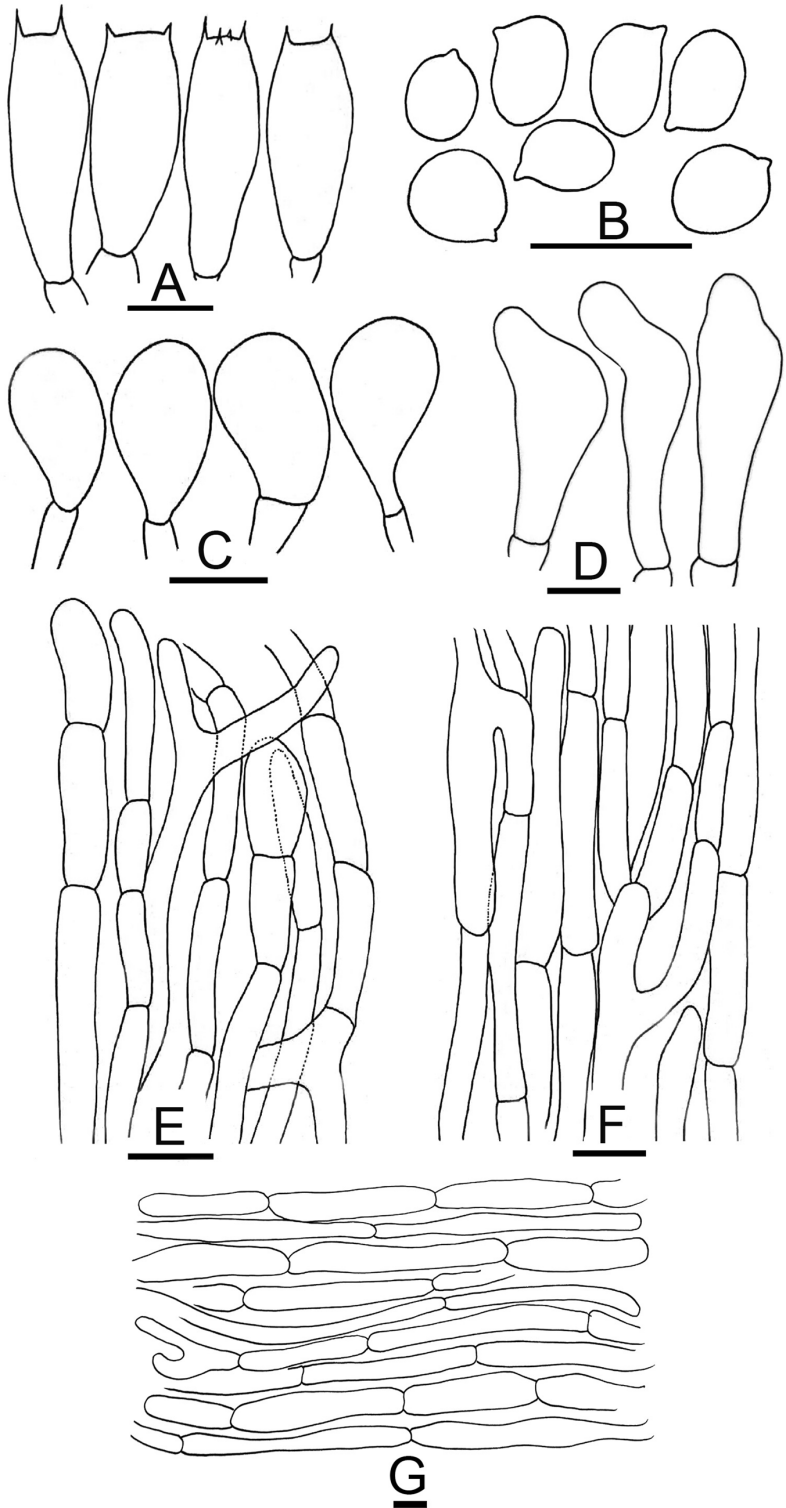
Micromorphological characters of *A.* *parviniveus.* **(A–C,E,F) **  B40 (**Holotype**); **(D) **  L20. (**A)** Basidia, **(B)** Basidiospores, **(C,D)** Cheilocystidia, **(E)** Pileipellis hyphae, **(F)** Stipitipellis hyphae, **(G)** Hyphae of the underside of the annulus. Bars = 10 µm. Drawings by Dr. Hira Bashir.

***Agaricus*** ***parviniveus*** H. Bashir & Khalid, *sp.* *nov*. Figs. 14A–F, 15A–G

MycoBank: MB828266

**Main** **characters:** This species is characterized by its small size, smooth (rarely squamulose) white pileus, stipe turning brown when rubbed, collar-like triple edged annulus, context white, unchanging when cut, odor pleasant not phenolic, basidiospores 5.6 × 4.3 µm on average and basidia frequently bisporic.

**Typification:** Pakistan, Punjab, University of the Punjab, Lahore, at 217 m a.s.l., solitary on rich loamy soil on the grounds of Botanical Garden, University of the Punjab, 1 Aug 2017, H. Bashir B40 (**holotype** LAH35358). GenBank: ITS = MK101030; LSU = MK100289; TEF1 = MK169411.

**Etymology:** The specific epithet derives from the two Latin words “*parvi*” meaning small and “*niveus*” referring to the white color of pileus.

**Species-specific** **ITS** **markers:** aggat[GGAT]ttgca@154-158, tctaaAcctat@276, tcgtc[A]ttcag@634 and ttgaaCgcttg@655.

**Original** **description:** *Pileus* 2.5–5 cm in diam., convex with incurved margin to plano-convex with straight margin at maturity, broadly umbonate only in collections (B40 and PU318), completely white (5.7 PB 8.4/2) or light ochraceous (5.4YR 5.6/1.6) at disc, smooth or with few subtle appressed concolorous or light brown (8.4YR 5.5/1.5) squamules. *Surface* dry and dull. *Margin* entire, sometimes radially split at maturity, strongly exceeding lamellae. *Lamellae* 2–4 mm broad, dark brown (7.5YR 3.3/1.9), free, crowded, intercalated with lamellulae and entire edges. *Stipe* 3–4 × 0.7 cm, cylindrical, clavate or slightly bulbous, stuffed, provided with an annulus in its upper part, smooth and white above and below the annulus, becoming brown when rubbed, no discoloration when cut. *Annulus* superous, thick, triple edged, broad but appressed to the stipe (what is better noticed when the sporocarp is cut longitudinally), collar–like, white to light brown (3.8YR 4.5/1.1). *Context* white, unchanging when cut. *Odor* pleasant, not phenolic.

*Basidiospores* (4.8–) 5.2–5.9 (–6.5) × (3.7–) 4.0–4.5 (–4.9) µm, [avX = 5.6 ± 0.67 × 4.3 ± 0.37 µm, Q_m_ = 1.28, n = 7 × 30], subglobose to ellipsoid, light to dark brown and some hyaline in KOH, smooth with a prominent apiculus, without apical pore and with granular content. *Basidia* 20–31 × 7.5–10 µm, hyaline in KOH, frequently bisporic rarely tetrasporic, clavate, truncate at the apex. *Cheilocystidia* abundant, simple, usually broadly clavate to pyriform, 12–18 × 5–10.5 µm, rarely fusiform (only in L20) 28.5–38 × 8.5–9.5 µm. *Pleurocystidia* absent. *Underside* *of* *the* *annulus* consisting of hyphae 3.5–11 µm in diam., hyaline in KOH, the broader the more constricted at septa, branched, terminal elements with rounded ends. *Pileipellis* consisting of cylindrical hyphae 5–8 µm in diam., frequently septate, branched, slightly constricted at septa, hyaline in KOH, terminal elements with rounded ends. *Stipitipellis* hyphae 4–7.5 µm in diam., cylindrical, parallel, branched, hyaline in KOH.

**Macrochemical** **reactions:** KOH reaction positive, light yellow. Schaeffer’s reaction negative.

**Habit,** **habitat** **and** **distribution:** Solitary on grassy grounds near *Dalbergia* *sissoo* Roxb. ex DC.*,* *Eucalyptus* *camaldulensis* Dehnh. and *Acacia* *nilotica* (L.) Delile trees. Until now, it is distributed from subtropical semi-arid areas of Lahore, Narowal and Toba Tek Singh to the arid area of Cholistan desert, Pakistan.

**Additional** **specimens** **examined:** Pakistan, Punjab, University of the Punjab, Lahore, at 217 m a.s.l., solitary on rich loamy soil on the grounds of the University, 1 Aug 2016, H. Bashir, PU248 (LAH35359). GenBank: ITS = MK101028; Punjab: University of the Punjab, Lahore, at 217 m a.s.l., solitary on rich loamy soil on the grounds of the University, 1 Sep 2016, H. Bashir, PU257 (LAH35360). GenBank: ITS = MK101029; Punjab: Toba Tek Singh, at 183 m a.s.l., solitary in grassy ground near *Dalbergia* *sissoo*, 26 Jul 2015, H. Bashir, TTS42 (LAH35361). GenBank: ITS = MK101031; PUNJAB: Lal Suhanra National Park, Cholistan desert, Bahawalpur, at 140 m a.s.l., scattered on sandy soil near *Acacia* *nilotica*, 30 Aug 2016, H. Bashir & M. Usman, L19 (LAH35362). GenBank: ITS = MK101026; PUNJAB: Lal Suhanra National Park, Cholistan desert, Bahawalpur, at 140 m a.s.l., scattered on sandy soil near *Acacia* *nilotica*, 30 Aug 2016, H. Bashir & M. Usman, L20 (LAH35363). GenBank: ITS = MK101027; Punjab: Narowal, at 375 m a.s.l., solitary on rich loamy soil on roadside, 14 Aug 2016, Humaira Bashir, NWL314 (LAH35364). GenBank: ITS = MK007251; Punjab: University of the Punjab, Lahore, at 217 m a.s.l., solitary on rich loamy soil on the grounds of the University, 1 Sep 2016, H. Bashir, PU318 (LAH35856). GenBank: ITS = MH997906.

**Notes:** Morphologically, *Agaricus* *parviniveus* is well characterized by having a thick, triple edged, broad annulus but which remains appressed to the stipe. Only four species in *A*. sect. *Xanthodermatei* have this type of annulus, *A.* *iodosmus* Heinem., *A.* *menieri* Bon, and sometimes *A.* *laskibarii* L.A. Parra & P. Arrill. and *A.* *parvitigrinus* Guinb. & Callac. *Agaricus* *iodosmus*, *A.* *laskibarii* and *A.* *menieri* differ by having much larger size, faint to strong yellow discoloration of the context when cut, a strong odor of phenol and much bigger spores. *Agaricus* *parvitigrinus* differs by having a slender habit, a pileus covered by brown squamules with entire darker centre, a context with yellow discoloration when cut, a faint odor of phenol and much narrower spores (3.73 µm on average).

**Figure 16:**
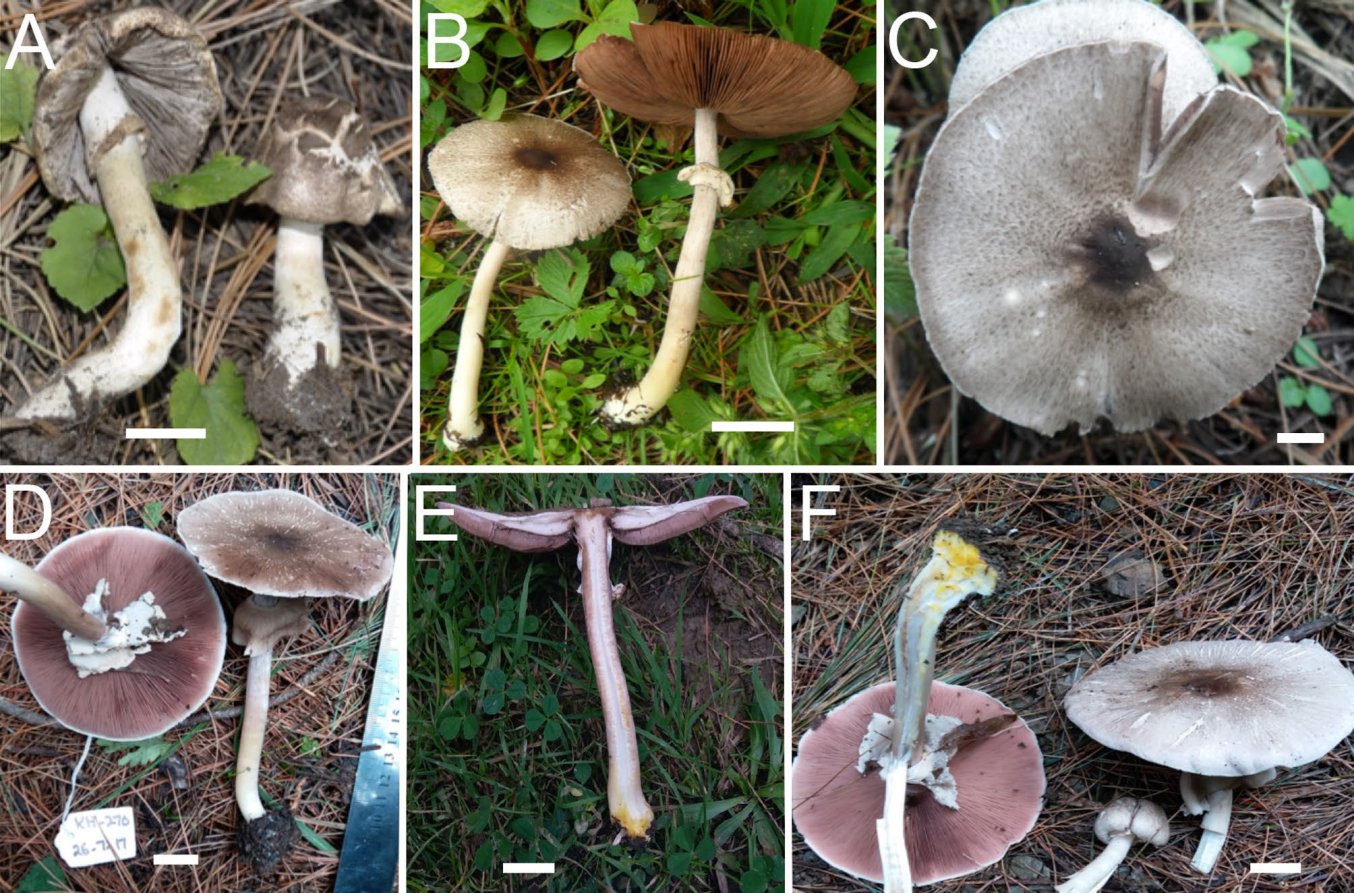
Macromorphological characters of *A.* *swaticus* **(A–C)** and *A.* *xanthochromaticus* **(D–F)**. **(A–F)** Basidiomata in the field. **(A)**  SJ60 (**Holotype**); **(B,C) **  SA111; **(D)**  KH262 (**Holotype**); **(E)**  KH270; **(F)**  KH295. Bars = 1 cm. Photographed by Dr. Hira Bashir.

**Figure 17:**
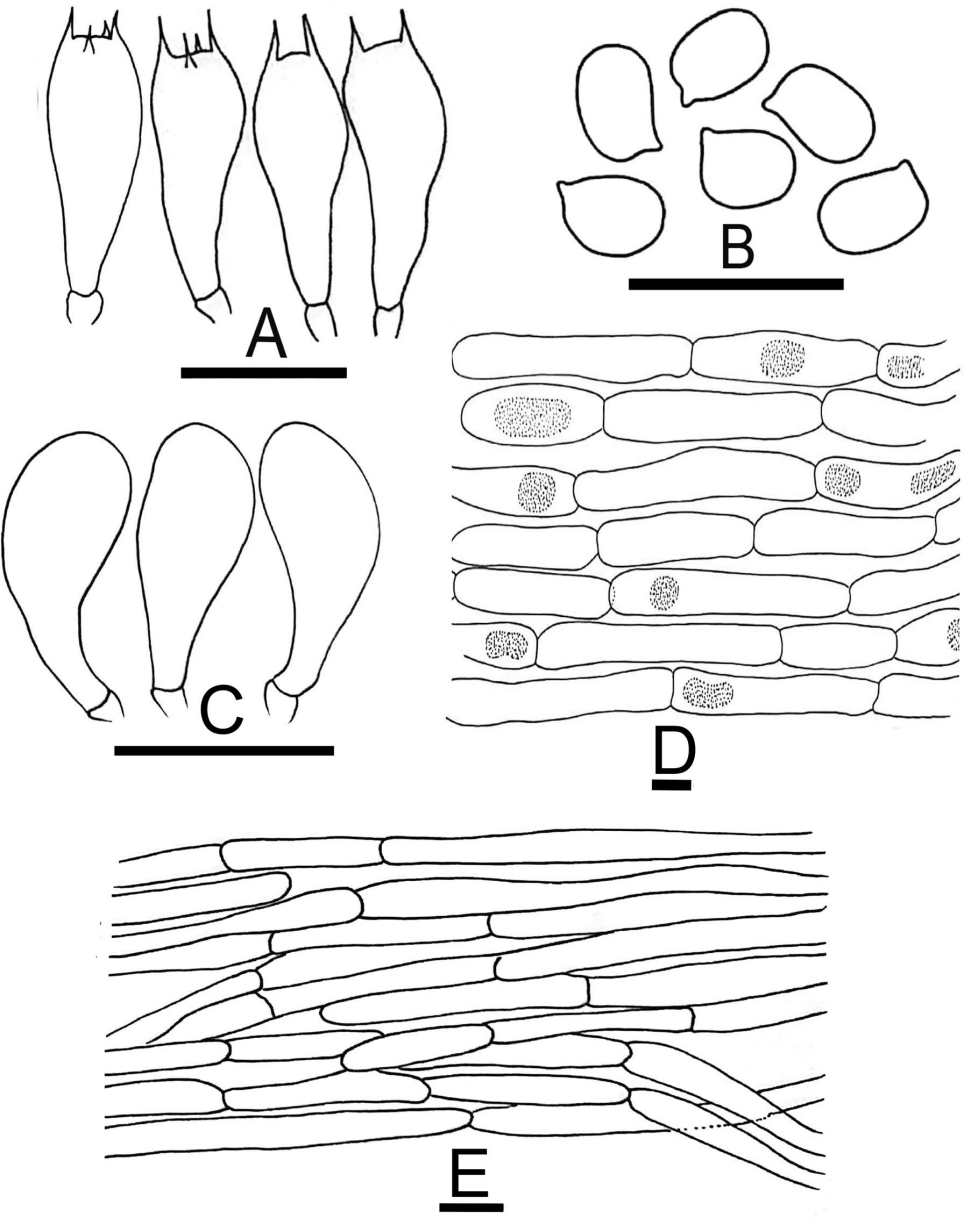
Micromorphological characters of *A.* *swaticus.* **(A–E)** SJ60 (**Holotype**). **(A)** Basidia, **(B)** Basidiospores, **(C)** Cheilocystidia, **(D)** Pileipellis hyphae, **(E)** Stipitipellis hyphae. Bars = 10 µm. Drawings by Dr. Hira Bashir.

***Agaricus*** ***swaticus*** H. Bashir, Jabeen, S. Ullah, Khalid & L.A. Parra, *sp.* *nov.* Figs. 16A–C, 17A–E

MycoBank: MB818953

**Main** **characters:** *Agaricus* *swaticus* is characterized by a large pileus, with grayish scattered scales condensed at the dark grayish brown umbonate centre, light grey to brown lamellae, basidiospores 6.1 × 4.0 µm on average and basidia bisporic to tetrasporic.

**Typification:** Pakistan, Khyber Pakhtunkhwa, Swat, Mashkun, at 2500 m a.s.l., solitary on ground under *Cedrus* *deodara* (Roxb.) Loud., 5 Sep 2013, S. Jabeen & A.N. Khalid, SJ60 (**holotype** LAH35199). GenBank: ITS = KY741895; LSU = KY741902.

**Etymology:** The specific epithet “*swaticus*” refers to the Swat district where this species was first collected.

Species-specific ITS markers—None.

**Original** **description:** *Pileus* 7.5–11 cm in diam., convex when young then plano-concave, umbonate at disc sometimes indistinctly umbonate. dark greyish brown (10YR3/4) entire at umbo, covered by dark greyish brown dotted squamules on a white (10YR9/2) background outside the umbo. *Surface* dry and dull, sometimes rimose (SJ60). *Margin* slightly incurved and wavy in young sporocarps, entire to radially ruptured at maturity, slightly exceeding the lamellae. *Lamellae* grey (in SJ60) (8.8YR 6.9/0.5) to brown (in SA111) (7.6YR 3.8/5.1), free, crowded, intercalated with lamellulae and entire edges. *Stipe* 4–10 × 0.5–1.3 cm, cylindrical to slightly bulbous at the base, stuffed, with rhizomorphs at the base, provided with an annulus in its upper part near lamellae, smooth, white (N10) above and below annulus, discoloring light brown when touched. *Annulus* superous, double-edged, thick, narrow, membranous, smooth on both sides, white becoming light brown with maturity. *Context* white when cut, discoloring faint yellow upon bruising. *Odor* of phenol, mild.

*Basidiospores* (5.5–) 5.6–6.7 (–7.3) × (3.5–) 3.7–4.1 (–4.7) µm, [avX = 6.1 ± 0.41 × 4.0 ± 0.31 µm, Q_m_ = 1.52, n = 3 × 30], broadly ellipsoid, light to dark olivaceous brown in KOH, smooth with a prominent apiculus and without apical pore. *Basidia* 10–17.5 × 5–8 µm, clavate, hyaline in KOH, bisporic to tetrasporic. *Cheilocystidia* observed only in the type collection (SJ60), 11–17 × 6–8.5 µm, broadly clavate, hyaline in KOH. *Pleurocystidia* absent. *Underside* *of* *the* *annulus* not observed. *Pileipellis* consisting of hyphae 5–11 µm in diam., frequently septate, cylindrical, some hyaline and others containing internal diffuse or vacuolar brown pigment in KOH, terminal elements with rounded tips. *Stipitipellis* constituted by hyphae 3.7–6.9 µm in diam., cylindrical, parallel, some hyaline and others light brown in KOH.

**Macrochemical** **reactions:** KOH reaction positive, light yellow. Schaeffer’s reaction negative on dry basidomata.

**Habit,** **habitat** **and** **distribution:** Growing solitary or gregarious on grassy places under or near *Cedrus* *deodara* (Roxb.) Loud. trees. Only known from the plains of southeast Pakistan so far.

**Additional** **specimens** **examined:** Pakistan, Khyber Pakhtunkhwa, Swat, Kalam, at 2060–2065 m a.s.l., solitary on ground under *Cedrus* *deodara*, 3 Sep 2013, S. Jabeen & A.N. Khalid, SJ53 (LAH35198). GenBank: ITS = KY741894; Shangla, at 850–2350 m a.s.l., solitary on grassy ground, 27 Jul 2014, Sadiq Ullah, SA111 (LAH35200). GenBank: ITS = KY741896.

**Notes:** Basidiomata of *A.* *swaticus* have been collected from different areas of Swat district. Morphologically some differences have been observed, possibly due to contrasting climatic conditions. Pileus surface rimose, umbonate, dull and dry, dark greyish brown, with incurved and wavy margin was noticed in the samples collected from the dry temperate regions (SJ53 and SJ60), and a fleshy pileus having brown scales on surface, indistinctly umbonate, slightly recurved and split margin in specimen SA111 collected from the moist temperate region. However, there are no molecular differences between the ITS sequences of the three specimens.

In our phylogenetic analyses *A.* *swaticus* is sister to *A.* *langensis* and is also phylogenetically related to *A.* *parvitigrinus,* and *A.* *californicus* Peck. The ITS sequences of *A.* *langensis* and *A.* *swaticus* differ at two positions from each other, but their LSU sequences also differ at one position, which is much more significant because this DNA region is much less variable than the ITS region. Morphologically *A.* *langensis* differs from *A.* *swaticus* by its white unchanging context when cut, its larger spores (7.2 × 4.4 µm on average) and the lack of cheilocystidia. The fact that both molecular and micro-morphological differences with *A.* *langensis* are consistent among our three specimens of *A.* *swaticus* collected in different locations, supports our taxonomic treatment of *A.* *swaticus* as a distinct species. However, the original description of *A.* *langensis* by He et al. (2018) was based on a single collection and therefore the morphological variability of *A.* *langensis* is still unknown.

*Agaricus* *parvitigrinus* differs by having a pileus with white margin, a narrow annulus, a faint odor of phenol, slightly smaller spores (5.83 × 3.73 µm on average) and basidioliform scarce cheilocystidia, and *A.* *californicus* by having a pileus with white margin, unchanging context when cut, and spores shorter and wider on average (5.8 × 4.3 µm) with a much lower Q coefficient (1.35).

**Figure 18:**
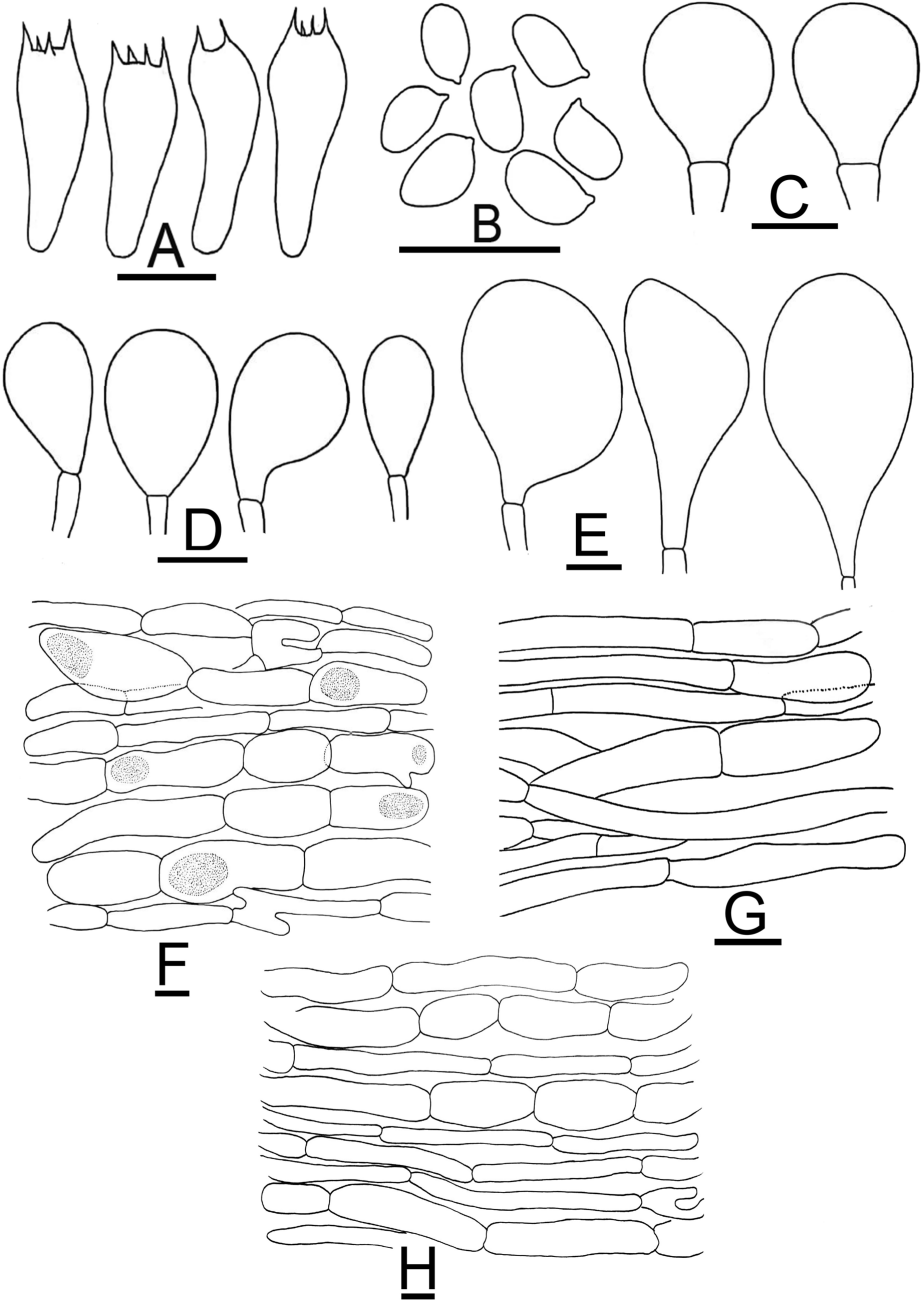
Micromorphological characters of *A.* *xanthochromaticus.* **(A–H)**  KH262 (**Holotype**). **(A)** Basidia, **(B)** Basidiospores, **(C–E)** Cheilocystidia, **(F)** Pileipellis hyphae, **(G)** Stipitipellis hyphae, **(H)** Hyphae of the underside of the annulus. Bars = 10 µm. Drawings by Dr. Hira Bashir.

***Agaricus*** ***xanthochromaticus*** H. Bashir, Khalid, L.A. Parra & Callac *sp.* *nov.* Figs. 16D–F, 18A–H

MycoBank: MB828315

**Main** **characters:** This species is characterized by a large pileus, with fine dot-like brown scales denser at disc, a long stipe having concolorous squamules to the pileus, a thick and flaring annulus, a chrome-yellow discoloration at the stipe base and basidiospores 4.9 × 3.3 µm on average.

**Typification:** Pakistan, Khyber Pakhtunkhwa, Helipad forest near Khanspur village, at 2250 m a.s.l., under *Pinus* *wallichiana* A.B. Jacks, gregarious, on decomposing needles, 26 Jul 2017, H. Bashir & M. Ali, KH262 (**holotype** LAH35365). GenBank: ITS = MK101034; LSU = MK100282; TEF1 = MK169404.

**Etymology:** The specific epithet derives from the Latinized Greek words: *xanthos* meaning yellow and *chroma* meaning color, referring to the chrome-yellow discoloration at stipe base when cut.

**Species-specific** **ITS** **markers:** None but the simultaneous presence of both markers ctcttGggagc@39 and gtcagTcttat@126 characterizes this species.

**Original** **description:** *Pileus* 8.5–13.5 cm in diam., broadly parabolic when young, convex to plano-convex at maturity, covered by brown (5.3YR 2.1/3.3) dotted squamules denser at disc, progressively scattered towards the margin on a white (10BG 9.9/0.5) background, somewhat radially fissured when completely expanded. *Surface* dry and dull. *Margin* entire slightly incurved in young sporocarps, wavy (in KH262, KH270), slightly exceeding the lamellae. *Lamellae* 3–5 mm broad, pinkish brown (4RP 6.6/3.5) to brown (3.7YR 2.1/4), free, crowded, intercalated with lamellulae and entire edge. *Stipe* 10–15 × 0.8–1.5 cm, 2.5–3.0 cm at the bulbous or abruptly bulbous base, provided with annulus in its upper part close to the lamellae, above the annulus pale pink to light brown (1.8YR 4/1.5), below the annulus white having some concolorous (0.3 PB 7.5/3.8) scattered floccose squamules towards the base which is usually provided with mycelial strands and discoloring rusty brown to dark brown when rubbed. *Annulus* superous, broad, thick, flaring, upper side white and subtly radially striate, underside white, conspicuously floccose, with light brown linear squames arranged as a cogwheel. *Context* white (N10), immediately chrome-yellow at the base when cut. *Odor* of phenol, strong.

*Basidiospores* (4.5–) 4.7–5.1 (–5.3) × (3.0–) 3.1–3.3 (–3.5) µm, [avX = 4.9 ± 0.37 × 3.3 ± 0.23 µm, Q_m_ = 1.48, n = 3 × 30], ellipsoid, some hyaline and others brown in KOH, smooth with a prominent apiculus. *Basidia* 14–23 × 6–7.5 µm, narrowly clavate, hyaline in KOH, mostly tetrasporic, some tri- or bisporic, smooth. *Cheilocystidia* abundant, usually simple, sometimes septate at the base, terminal element 10–20 × 8–17 µm (KH262, KH295) or bigger 40–46.5 × 20–27 µm (KH270), broadly clavate, pyriform, globose or sphaeropedunculate, ante-terminal elements shorter and cylindrical. *Pleurocystidia* absent. *Underside* *of* *the* *annulus* consisting of cylindrical hyphae, 4–8.5 µm, hyaline in KOH, the wider the more shorter and constricted at septa, terminal elements rounded. *Pileipellis* hyphae 4.5–13 µm in diam., frequently septate, branched, the wider the more constricted at the septa, in KOH some hyaline and some with an internal brown vacuolar or diffuse pigment in KOH, terminal elements with rounded ends. *Stipitipellis* hyphae 3–8.5 µm in diam., cylindrical, parallel, hyaline, rarely branched.

**Macrochemical** **reactions:** KOH reaction positive, bright yellow. Schaeffer’s reaction negative.

**Habit,** **habitat** **and** **distribution:** Growing in clusters on decomposing needles of pine trees in pine dominating moist temperate forest of Pakistan.

**Additional** **specimens** **examined:** Pakistan, Khyber Pakhtunkhwa, Helipad forest near Khanspur village, at 2250 m a.s.l., under *Pinus* *wallichiana*, gregarious, on decomposing needles, 26 Jul 2017, H. Bashir & M. Ali, KH295 (LAH35367). GenBank: ITS = MK101036; LSU = MK100283; TEF1 = MK169405; Khyber Pakhtunkhwa, Ayubia National park, at 2400 m a.s.l., under *Pinus* *wallichiana*, gregarious, on decomposing needles, 30 Jul 2017, H. Bashir & M. Ali, KH270 (LAH35366). GenBank: ITS = MK101035;

**Notes:** Morphologically, an unequivocal morphological differentiation with all taxa of *A*. subsect. *Xanthodermatei* is not possible. Phylogenetically, the most closely taxa are *A.* *griseovariegatus* and *A.* *sinoplacomyces*. *Agaricus* *griseovariegatus* differs by having an unchanging context when cut at stipe base and *A.* *sinoplacomyces* differs by having slightly longer spores (5.3 µm on average) and by lacking cheilocystidia.

### Brief description of five species of *A*. sect. *Xanthodermatei* based on new specimens

Three of them were already reported from Pakistan, one is newly reported from Europe, and the remaining one is a putative new species from Caribbean

**Figure 19:**
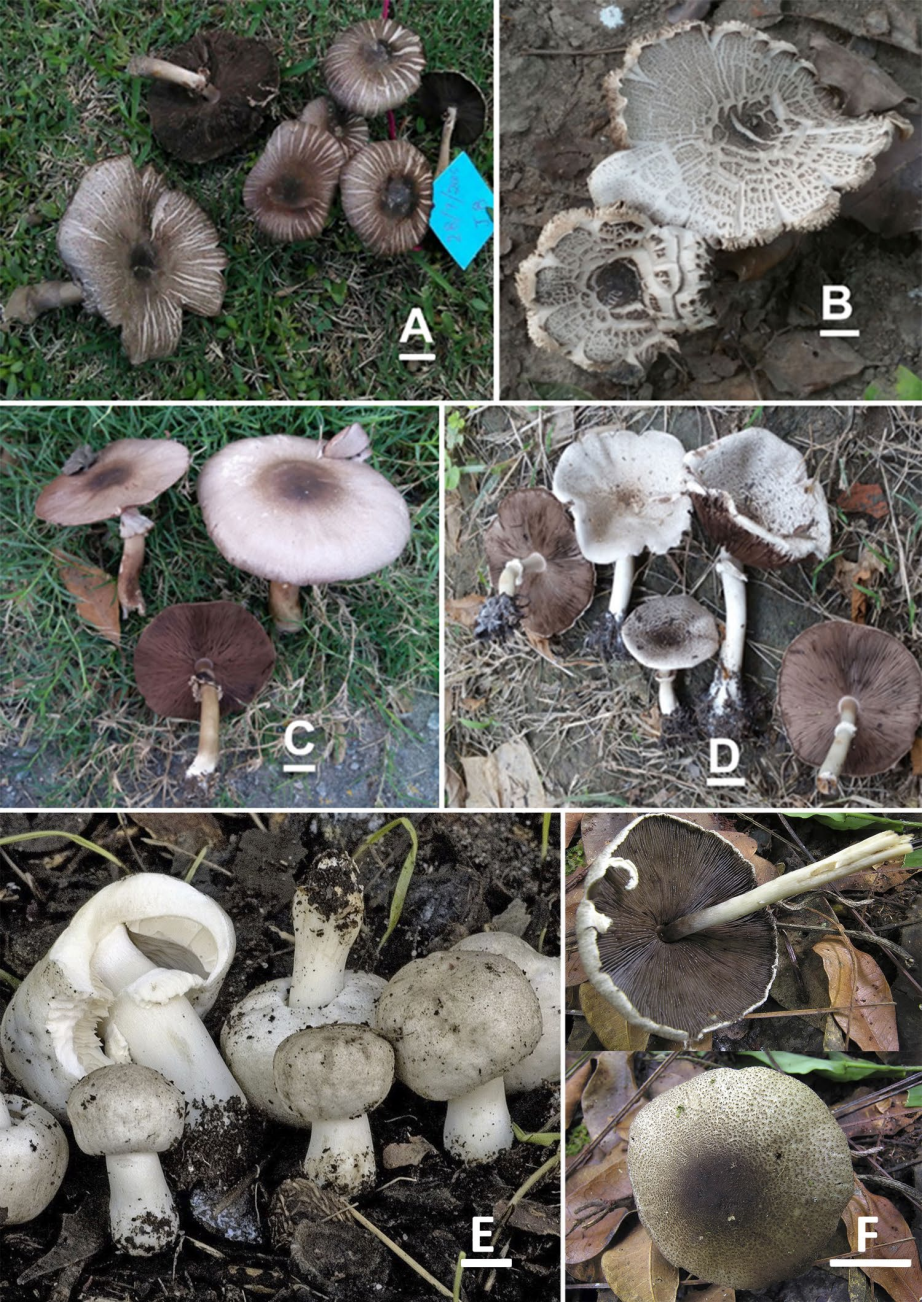
New collections of species either previously reported from Pakistan **(A–D)**, newly reported from Spain **(E)**, or remaining unnamed from Dominican Republic **(F)**. Basidiomata in the field. **(A)**  J8 (*A.* *endoxanthus*), **(B) **  L11 (*A.* *punjabensis*), **(C)**  CM200 (*A.* *punjabensis*), **(D)**   PU252 (*A.* *bisporiticu*s), **(E) **  LAPAG 608 (*A.* *californicus*), **(F) **  JBSD127423/LAPAM 110 (*A.* sp.). Bars = 1 cm. Photographed by **(A–D)** Dr. Hira Bashir, **(E)** Enrique Rubio, **(F)** Claudio Angelini.

***Agaricus*** ***bisporiticus*** Nawaz, Callac, Thongkl. & Khalid, in Thongklang, Nawaz, Khalid, Chen, Zhao, Parra, Hanif, Moinard & Callac, *Mycologia* 106(6): 1224 (2014). Fig. 19D

MycoBank: MB808224

A new specimen (PU252, Fig. 19D) of this species of *A.* sect. *Xanthodermatei* (clade Xan II) was collected in Lahore, which is in a subtropical region of Pakistan. Morphological, molecular and phylogenetic data completely agreed with those previously reported for samples of this species from Pakistan and Thailand by Thongklang et al. (2014). We have examined also the type of *A.* *bisporiticus* (MCR25) provided by LAH herbarium and our observations agree with those published in the original description since bisporic, trisporic, and tetrasporic basidia were observed frequently, spores are rather similar, measuring (4.5–) 5.7–6.9 (–7.5) × (3.0–) 3.5–4.2 (–5.0) µm.

***Agaricus*** ***endoxanthus*** Berk. & Broome, J. *Linn.* *Soc.,* *Bot.* 11: 548 (1871) Fig. 19A

MycoBank: MB226162

A new specimen (J8, Fig. 19A) of this species of *A*. sect. *Xanthodermatei* (clade Xan III) was collected from Jallo Park, Lahore, Pakistan. Phylogenetic analyses and morphological observation confirm the identification of this cosmopolitan tropical and subtropical species previously collected in Punjab, Pakistan (Chen et al. 2016). As in the specimen from Punjab, the new specimen typically exhibits a radially cracked dark greyish pileus surface and a chrome yellow discoloration at stipe base immediately when cut.

***Agaricus*** ***punjabensis*** T. Qasim, A. Ashraf & Khalid, Phytotaxa 252 (1): 8 (2016) Fig. 19B,C

MycoBank: MB814981

Three new specimens (L11, PU265 and CM200; Fig. 19) of this species of *A*. sect. *Xanthodermatei* (clade Xan III) were collected from Changa Manga forest (CM200) and Lahore (L11 and PU265). With three polymorphic ITS-nucleotide sites, and high morphological variability, the subtropical species *A*. *punjabensis* appears to be quite variable (Fig. 19). For example, the pileus of the specimen PU265 is fissured in an irregular pattern forming various small patches of dark brownish grey squames. The pileus diameter of CM200 is bigger (11.5 cm) than those described by Chen et al. (2016). In the previous report of *A.* *punjabensis*, the discoloration was not recorded. A bright yellow discoloration and a strong odor of phenol at stipe base was noted in our new collections.

***Agaricus*** ***californicus*** Peck, *Bull.* *Torrey* *bot.* *Club* 22: 203 (1895) Fig. 19E

MycoBank: MB220273

A new specimen (LAPAG 608; GenBank MK215826) of this species of *A*. sect. *Xanthodermatei* (clade Xan III) was collected from Navia, Asturias, Spain. This specimen constitutes the first record for this species in Europe, and outside North America where it was described for the first time. Phylogenetic analyses confirmed its 100% identity of its ITS1 + 2 region with the specimen RWK 1914 (GenBank DQ182509) from Monterey, California, USA.

It must be noted that another ITS sequence of this sample (called RWK1914) with GenBank accession number AY464879 is a wrong sequence. Indeed, the ITS1 region is similar to the sequence of RWK 1914/DG182509, while the ITS2 region is similar to a sequence of *A.* *porphyrocephalus* (RWK 2088/KJ877758). The morphological characters of the Spanish specimen match very well with those published by Kerrigan (2016) and macroscopically is very similar to the Californian specimen RWK 1072. Microscopically, LAPAG 608 also has an average spore size very similar (5.6 × 4.17 μm; Q = 1.34) to Kerrigan’s measurements for this species (5.8 × 4.3 μm; Q = 1.35). The cheilocystidia and basidia are also very similar in shape and size, but, in addition, LAPAG 608 also possesses scattered large-sized cheilocystidia (22–42 × 15–32 μm), pleurocystidia (21–32 × 15–20 μm) and cystidiform hyaline macrobasidia (19–35 × 10–18 μm).

***Agaricus*** **sp.** Fig. 19F.

A specimen (JBSD127423, duplicate in LAPAM 110) belonging to *A.* sect. *Xanthodermatei* (clade Xan II) was collected in a mixed forest with broadleaf trees and *Pinus* *occidentalis* in Jarabacoa, La Vega, Dominican Republic. This specimen belongs to a new species which is not formally named because only a single incomplete basidiomata, lacking the stipe base, was collected. This specimen is remarkable for its pileus surface with olivaceous tones, a color never noticed in any member of clade Xan II. Pileus 3 cm in diameter, hemispherical, dark grayish-brown with olivaceous tones, entire at center and covered elsewhere by small scales, punctiform near the center and triangular towards the margin on a whitish background, with margin slightly exceeding the lamellae. Annulus attached to the pileus margin, 2 mm broad, with scales in the lower margin. Lamellae 3 mm broad, dark brown with whitish edge. Stipe 3 mm wide, smooth, whitish with a brown apex. Context white, with a very faint yellow discoloration towards the base when broken, without a distinctive odor.

## Discussion

### A brief overview on the relationships between *A.* sect *Hondenses* and *A.* sect. *Xanthodermatei*

This study focuses on *A.* sect. *Xanthodermatei* and *A.* sect. *Hondenses*. These two sections share morphological characteristics, which allow to distinguish them from the other eleven sections in *A*. subg. *Pseudochitonia*. In recent studies of Zhao et al.^7^ and Parra et al.^4^, *A.* sect. *Hondenses* appears closer to *A.* sect. *Bivelares* than to *A.* sect. *Xanthodermatei* in multi-gene trees but without support value. In contrast, *A.* sect. *Hondenses* and *A.* sect. *Xanthodermatei* are sister to each other in Zhou et al.^22^, and in the present study with a relatively good support (BS = 78, PP = 1). This relationship will have to be confirmed in the future in a larger multi-gene analysis including the 13 sections of the subgenus and a higher proportion of samples with multi-gene data. However, it should be noted that, for example, the number of species of these two sections with TEF1 sequence data already increased from 9 in Zhao et al.^7^ to 29 in the present study.

### Comparison of the major clades and interest of indel markers

Despite our efforts to compare the clades Xan I, Xan II, and Xan III, we did not find any morphological feature reliably characterizing these clades. According to Table 1, temperate species are clearly preponderant in Xan I (7/8) and Xan III B (8/11), while tropical or subtropical species are preponderant in Xan II (11/16) and Xan III A (1/1). The clade Xan IIIC is complex and include the half of all the named or unnamed species (36/72) with some remarkable subclades. For example, a well-supported subclade includes four (sub)tropical species of which three are found in Pakistan (*A.* *endoxanthus*, *A.* *fumidicolor* and *A.* *punjabensis*), while another subclade comprises four temperate species including the type species of the section *A.* *xanthodermus*.

Comparing the ITS barcode sequences of all the species, we report three discriminating markers between the three clades Xan I, Xan II and Xan III. Two of them are clade-specific indels, which are sufficient to classify all the species in the three clades. A risk when using clade or taxon-specific marker is that its specificity can be lost when new species are discovered. It is what happened for certain clade-specific markers previously reported by Thongklang et al.^18,19^. Apparently, indel markers remained more reliable than nucleotide markers possibly due to a lower homoplasy^49^. In addition, such indels are phylogenetically informative and could be included in the analyses, however for our current purpose this was not necessary since even without them, the major clades were phylogenetically well-supported in our multi-gene tree. Nevertheless, in the tree only based on ITS sequences (Fig. 2), the sections and major clades are generally present and as well- or less well-supported than in the multi-gene tree with the exception of Xan II and Xan III A. Indeed, Xan II appears as a paraphyletic group and, in addition, the single species of clade Xan III A (*A.* *flavidodiscus*) is mixed with species of the clade Xan II. In fact, using only ITS sequence, the placement of *A.* *flavidodiscus* is unstable. For example, it appeared in Xan III in both ITS and multigene trees of Parra et al. (2018) but this was well-supported only in the multigene tree. Phylogeny of clades Xan II and Xan III A remains improperly resolved in ITS tree.

In conclusion, multi-gene analyses were necessary to establish the major clades and their phylogeny. However, two indels in ITS alignment are efficient to classify the species in the three major clades. Despite Zhao et al.^7^ proposed the taxon *A*. sect. *Hondenses* for the clade Xan I, we did not try to propose taxa for the other clades, considering that it is presently not possible to reliably characterize them morphologically.

### Low level of intra- and interspecific variability in ITS sequences of *A*. sect. *Xanthodermatei*

We were surprised by the absence of variability among the ITS sequences of seven of the eight new species proposed in this study. Since this is generally not so frequent in genus *Agaricus*, we decided to examine on the one hand the intraspecific variability in *A.* *xanthodermus*, which is the type of *A.* sect. *Xanthodermatei* and one of the most documented species of this section in GenBank, and on the other hand the intra- and interspecific variabilities in a group of ten closely related species.

In the case of *A.* *xanthodermus*, we found that the 18 ITS sequences retrieved from GenBank and which are from three different continents are identical with the exception of a difference at a single nucleotide site in only one of them. Since we observed this low variability in most of or new species, one conclusion is that the intraspecific variability in the ITS sequences is frequently low in *A*. sect. *Xanthodermatei*. However, it is not a rule since, for example, ITS sequences in *A.* *endoxanthus* are highly variable^19^.

In the case of the group of closely related species (Table 2), we found that the intraspecific variability was quasi-absent in most of these species and that certain species differ from each other at a single ITS nucleotide site. This is the case of *A.* *deardorffensis* from North America and *A.* *tibetensis* from Asia. More surprisingly, this is also the case for *A.* *xanthochromaticus* and *A.* *griseovariegatus* which are both new species described from Pakistan. Such a low level of interspecific variability has been previously observed in this section for example between ITS sequences of *A.* *xanthodermus* and *A.* *moelleri* Wasser, which differ from each other at only at two positions^16^. We can also note that the new species *A.* *swaticus* and the recently described species *A.* *langensis* differ also at two positions from each other. It is noteworthy that in genus *Agaricus* when a single difference is observed between ITS sequences of two samples, they are generally considered as belonging to the same species. However, when several specimens are available and when this difference is correlated with morphological differences and/or, with differences in other sequenced genes, the two divergent entities are considered as distinct species. This is the case for the two new species *A*. *xanthochromaticus* and *A.* *griseovariegatus*, which not only are morphologically relatively well-distinguishable but also differ at 6 positions of their TEF1 sequences. This suggests that an efficient process of concerted evolution occurred for ITS. In other respects, contrarily to other groups of closely related species such as in *A.* sect. *Arvenses* (Konrad & Maubl.) Konrad & Maubl., there is no indication of reticulate evolution of hybridization between species or distant populations. This suggests that efficient reproductive barriers take place during the speciation process. Geographical and climatic factors may have contributed to these speciation events since these species are distributed in North America or in Asia and only one of them adapted to subtropical conditions (*A.* *sinoplacomyces*). However, three of them are temperate and allopatric species newly described (*A.* *griseovariegatus*, *A.* *macropeplus* and *A.* *xanthochromaticus*).

In conclusion, in *A.* sect. *Xanthodermatei*, we hypothesize that efficient reproductive barriers take place in speciation process and that efficient concerted evolution frequently occurs for ITS. We frequently observe a low level of intraspecific variability and a low level of interspecific variability between closely related species. Samples that differ at only one position in the ITS sequence might belong to different species more frequently than in other sections. In this case, it is prudent to have several collections and, as far as possible, to compare them with other markers than ITS like TEF1.

### Some unexpected features

In *A.* sect. *Hondenses*, the new species *A.* *bambusetorum* described with its smooth white small sized cap, its narrow simple annulus, its pleasant odor and its distribution in subtropical climatic area, is completely atypical in this section. In other respects, it was surprising to find bisporic basidia, sometimes abundantly, in all the new species. They were also observed in the new collection of *A.* *gregariomyces* and were abundant in the type as in the new collection of *A.* *bisporiticus*. Before interpreting these unexpected observations, it should be necessary to observe fresh specimens on dry microscopic preparation without cover glass to better estimate the proportions of n-spored basidia, not only for the species collected in Pakistan but for all the species of *A.* sect. *Xanthodermatei* in the future. Variable proportion of bisporic basidia are reported in different taxa of *A.* sect. *Bivelares*, which is closely related to *A.* sect. *Xanthodermatei*, such as in *A.* *bisporus*^50^ and more recently in *A.* *sinodeliciosus* Z.R. Wang & R.L. Zhao^51^. The proportion of bisporic basidia could be misestimated due to non-representative or too small sampling in dried specimens. In addition, this trait can be sensitive to environmental factors^52^. Spores of bisporic basidia could be heterokaryotic and in this case the life cycle is pseudohomothallic, which could strongly impact the population structure of these species and their evolution.

### A summary of the increase of the number of species and of the species richness in Pakistan

The number of named species with available ITS data set currently reaches 8 in *A*. sect. *Hondenses* and 53 in *A*. sect. *Xanthodermatei.* Among these 61 species, 16 were described before 2000, 33 from 2000 to 2018 and listed in^2^ with one correction (*A.* *freirei* Blanco-Dios must be placed in *A*. sect. *Hondenses* instead of *A*. sect. *Xanthodermatei*), 3 in 2019^35,53^, and 8 in this study. All 61 species were included in our analyses except the most recently described species *A.* *rubripes* J.F. Zheng & L.H. Qiu (holotype K17071201; ITS/GenBank MH220318; Guangdong Province China). For phylogenetic purpose, it was not necessary to update our analyses with this species because its ITS sequence differs at only one nucleotide site from those of *A.* sp./ZRLWXH3092, which was already included in the analyses and placed in clade Xan II (Figs. 1 and 2).

Among the 61 species identifiable with ITS barcode in both sections, 12 are reported from Pakistan including 8 new species and a new report from this country in the present study. This represents a high species richness compared to the only four species reported from Iran (Mahdizadeh et al. 2017) in the two sections, which, at this time, were still grouped in *A.* sect. *Xanthodermatei*. Species reported from Iran are all temperate and previously reported from Europe. In contrast, 9 of the 12 species found in Pakistan are endemic, only two are reported from other Asian countries (China and Thailand), and the remaining one is the cosmopolitan tropical species *A.* *endoxanthus*. None of these 12 species are known in Europe except occasional cases of introduction of *A.* *endoxanthus* in glass-houses. Half of them are tropical or subtropical and they are distributed in the different major clades as follows: 1 in Xan 1, 2 in Xan II, 9 in Xan III of which 2 are in Xan III B and 7 in Xan III C. This remarkable biodiversity can be partly explained by the diversity of the climates and biotopes in Pakistan.

For the sake of completeness 14 species are described. Eight new species and one new record for Pakistan are described in detail. New collections of three other species previously reported from Pakistan are briefly described. The two remaining brief descriptions do not concern samples from Pakistan but (i) a sample from Spain which formally represents the first report in Europe and outside North America of *A.* *californicus*, (ii) a sample of an unnamed species from Dominican Republic that we considered useful for its contribution to the clade Xan II in the phylogenetic analysis.

We predict that the number of species will continue to quickly increase and we recommend, as much as possible and more specifically for *A.* sect. *Xanthodermatei*, to use several specimens, to get TEF1 sequences, and to use the BLAST but with caution. Indeed, the percentage of identity can be incorrect due to heteromorphisms and many sequenced samples are misidentified in GenBank. In other respects, discoloration, odor and structure of the annulus should be carefully examined and reported from specimens in the field and it must be never forgotten that the species of these two sections are presumably poisonous.
